# Indication-Specific Opioid Prescribing for US Patients With Medicaid or Private Insurance, 2017

**DOI:** 10.1001/jamanetworkopen.2020.4514

**Published:** 2020-05-11

**Authors:** Christina A. Mikosz, Kun Zhang, Tamara Haegerich, Likang Xu, Jan L. Losby, Arlene Greenspan, Grant Baldwin, Deborah Dowell

**Affiliations:** 1 National Center for Injury Prevention and Control, Centers for Disease Control and Prevention, Atlanta, Georgia

## Abstract

**Importance:**

Although opioids can be effective medications in certain situations, they are associated with harms, including opioid use disorder and overdose. Studies have revealed unexplained prescribing variation and prescribing mismatched with patient-reported pain for many indications.

**Objective:**

To summarize opioid prescribing frequency, dosages, and durations, stratified across numerous painful medical indications.

**Design, Setting, and Participants:**

Retrospective cross-sectional analysis of 2017 US administrative claims data among outpatient clinical settings, including postsurgical discharge. Participants had any of 41 different indications associated with nonsurgical acute or chronic pain or postsurgical pain or pain associated with sickle cell disease or active cancer and were enrolled in either private insurance (including Medicare Advantage) in the OptumLabs Data Warehouse data set (n = 18 016 259) or Medicaid in the IBM MarketScan Multi-State Medicaid Database (n = 11 453 392). OptumLabs data were analyzed from October 2018 to March 2019; MarketScan data were analyzed from January to April 2019.

**Exposures:**

Nonsurgical acute or chronic pain or postsurgical pain; pain related to sickle cell disease or active cancer.

**Main Outcomes and Measures:**

Indication-specific opioid prescribing rates; days’ supply per prescription; daily opioid dosage in morphine milligram equivalents; and for chronic pain indications, the number of opioid prescriptions.

**Results:**

During the study period, of 18 016 259 eligible patients with private insurance, the mean (95% CI) age was 42.7 (42.7-42.7) years, and 50.3% were female; of 11 453 392 eligible Medicaid enrollees, the mean (95% CI) age was 20.4 (20.4-20.4) years, and 56.1% were female. A pain-related indication under study occurred in at least 1 visit among 6 380 694 patients with private insurance (35.4%) and 3 169 831 Medicaid enrollees (27.7%); 2 270 596 (35.6% of 6 380 694) privately insured patients and 1 126 508 (35.5% of 3 169 831) Medicaid enrollees had 1 or more opioid prescriptions. Nonsurgical acute pain opioid prescribing rates were lowest for acute migraines (privately insured, 4.6% of visits; Medicaid, 6.6%) and highest for rib fractures (privately insured, 44.8% of visits; Medicaid, 56.3%), with variable days’ supply but similar daily dosage across most indications. Opioid prescribing for a given chronic pain indication varied depending on a patient’s opioid use history. Days’ supply for postoperative prescriptions was longest for combined spinal decompression and fusion (privately insured, 9.5 days [95% CI, 9.4-9.7 days]) or spinal fusion (Medicaid, 9.1 days [95% CI, 8.9-9.2 days]) and was shortest for vaginal delivery (privately insured, 4.1 days [95% CI, 4.1-4.1 days] vs Medicaid, 4.2 days [95% CI, 4.2-4.2 days]).

**Conclusions and Relevance:**

Indication-specific opioid prescribing rates were not always aligned with existing guidelines. Potential inconsistencies between prescribing practice and clinical recommendations, such as for acute and chronic back pain, highlight opportunities to enhance pain management and patient safety.

## Key Points

**Question:**

What are indication-specific opioid prescribing rates for nonsurgical acute or chronic pain or postoperative pain conditions and pain related to cancer or sickle cell disease?

**Findings:**

In this cross-sectional analysis examining data from 18 016 259 patients with private insurance and 11 453 392 patients with Medicaid benefits, opioid prescribing rates, days’ supply, and daily dosage varied widely across clinical indications and by insurance type.

**Meaning:**

Potential inconsistencies between indication-specific prescribing patterns and relevant clinical recommendations highlight opportunities for selection of safer and more effective pain treatment options.

## Teaser

This cross-sectional study uses administrative claims data for outpatient clinical settings, including postsurgical discharge, from patients with private insurance or Medicaid beneficiaries to summarize opioid prescribing frequency, dosages, and durations for numerous painful medical indications.

## Introduction

Opioids can be essential drugs for pain management but carry significant risk for harm. In 2017, more than one-third of the 47 600 opioid overdose deaths in the US involved prescription opioids.^1^ In 2018, approximately 1.7 million Americans aged 12 or older struggled with prescription opioid use disorder (OUD).^2^ Although an increasing proportion of people entering OUD treatment report initiating opioid use with heroin, a majority report that their problematic use began with prescription opioids.^3^ Longer duration of opioid therapy is associated with increased OUD risk,^4^ and higher dosages are associated with increased overdose risk.^5^ Harms extend beyond patients with prescriptions—a majority of people misusing prescription opioids report obtaining them from friends or family with prescriptions.^2^

Guidelines provide recommendations to help clinicians and patients determine when the benefits of opioids might outweigh the risks and inform dosage and duration.^6,7,8^ However, there is variation in opioid prescribing practices across indications that does not match the level of evidence for treatment effectiveness. For example, chronic pain indications are the most common indications for which opioids are prescribed, despite evidence being insufficient to demonstrate long-term benefit of opioids for chronic pain.^6^ Furthermore, prescribing characteristics, such as initial days’ supply, have been associated with the likelihood of continued opioid use regardless of clinical factors such as pain etiology.^9^ Opioid prescribing has been shown to vary even across similar patients and indications within the same institution.^10^ Unexplained prescribing variation and reports that patients often use only a fraction of opioids prescribed postoperatively^11^ suggest there are opportunities to reduce unnecessary opioid prescribing.

At the same time, there have been reports of undertreatment or delay in pain treatment in instances in which the benefits of opioids might outweigh the risks.^12,13,14^ For example, a recent systematic review found that approximately one-third of cancer patients did not receive sufficient pharmacologic pain therapy,^12^ and several studies document delays and barriers among patients with sickle cell disease (SCD) receiving analgesics, including opioids.^13,14^

The objective of the present study was to estimate rates and amounts of opioids prescribed for specific indications to outpatients in the United States. Findings can provide greater insight into current prescribing practices, prescribing variance across indications and populations, and indications for which amplification of recommendations could improve pain management and patient safety.

## Methods

### Data Sources and Study Sample

We conducted a retrospective cross-sectional analysis of national deidentified administrative claims data from OptumLabs Data Warehouse (OLDW)^15^ and MarketScan Multi-State Medicaid Database (MMD). The OLDW covers 14% of the commercially insured population and 21% of the Medicare Advantage (collectively, “privately insured”) population throughout the US in 2016 and 2017. The OLDW population is nationally representative of privately insured patients with regard to age and sex. It covers all census regions although it overrepresents the South and Midwest. The MMD data set contains all Medicaid beneficiaries (fee for service and managed care) enrolled between 2016 and 2017 in 9 anonymized states distributed across census regions. This study adheres to the Strengthening the Reporting of Observational Studies in Epidemiology (STROBE) reporting guideline and the RECORD (REporting of studies Conducted using Observational Routinely-collected Data (RECORD) guideline for cross-sectional studies. The CDC National Center for Injury Prevention and Control determined that the research was conducted with existing data without individual identifiers; thus, the activity is research but does not involve human subjects and therefore institutional review board approval was not required.

The study period for identifying any index diagnosis was from January 1, 2017, to December 31, 2017, for OLDW and from October 1, 2016, to September 30, 2017, for MMD. Additional details on data sources, access, and sampling can be found in eAppendix 1 in the Supplement.

### Identification of Pain-Related Indications and Associated Opioid Prescriptions

We identified 41 different indications associated with pain, including acute and chronic conditions, surgical procedures, SCD, and cancer. Lists of indications, their selection criteria, and their associated *International Statistical Classification of Diseases, Tenth Revision, Clinical Modification* diagnosis and *Current Procedural Terminology* codes are described in eAppendix 1 and eAppendix 2 in the Supplement.

We identified opioid prescription claims during the study period using previously compiled National Drug Codes.^16^ We calculated daily morphine milligram equivalents (MME) for opioid prescriptions using days’ supply and quantity dispensed from opioid claims and MME conversion factors.^16^ We developed algorithms to link patients’ opioid prescriptions to their medical encounters (“visits”) for each indication using data such as patient identification, visit date, dispensing date, clinician identification, and index diagnosis (see eAppendix 1 in the Supplement for both written and visual descriptions of this linking algorithm). For acute pain indications and surgical procedures, the index diagnosis represents primarily the first appearance of the indication. However, for chronic pain indications as well as for SCD and cancer, the index diagnosis captured in our analysis represents both preexisting indications (for which patients may or may not have received opioids previously) as well as new indications (the first instance of an indication that becomes chronic) (eAppendix 1 in the Supplement).

### Expert Consultation

The Centers for Disease Control and Prevention (CDC) presented the research protocol and incorporated recommendations from the National Center for Injury Prevention and Control Board of Scientific Counselors, a federal advisory committee, based on expert input from the Board of Scientific Counselors Opioid Prescribing Estimates Workgroup (see eAppendix 1 in the Supplement for detailed discussion of the clinical specialties that comprised this expert Workgroup; the Workgroup’s series of meetings and resulting report to the Board of Scientific Counselors; and a full roster of Workgroup members).

### Statistical Analysis

For nonsurgical acute or chronic pain or postsurgical pain indications, we calculated the number of visits, patients, or procedures, respectively, associated with each indication; prescribing rate for each indication, overall and by age group (0-18 years, 19-64 years, and ≥65 years); the mean with its 95% CI and the median with its interquartile range (IQR) of daily opioid dosage in MME; and the mean (95% CI) and median (IQR) days of opioids supplied. For SCD and cancer, we calculated prescribing rates overall and by age group. For postsurgical and nonsurgical acute pain indications, which generally represent individual events at a single point in time, reported outcome data (prescribing rate, MME, and days’ supply) reflect prescriptions supplied for a specific procedure or visit, meaning that the prescribing rate is anchored to visits or procedures. For chronic pain indications, SCD, and cancer, for which a single patient may receive multiple prescriptions for the same indication over time, reported outcome data (prescribing rate, MME, days’ supply, and number of prescriptions) are anchored to patients, reflecting all prescriptions supplied to a specific patient for visits related to that indication during the 3 months following the index diagnosis. Prescribing measures among chronic pain and postsurgical indications were stratified by opioid use history. Long-term opioid therapy (LTOT) was defined as having (1) 3 or more opioid prescriptions in the 3 months prior to the index visit; (2) more than 60 total days of opioid supply; and (3) a gap between the end of one prescription and the next prescription of less than 10 days. Patients not receiving LTOT were those whose prescriptions did not meet LTOT criteria. Only patients not receiving LTOT were included in the nonsurgical acute pain analysis to increase confidence in opioid prescription linkage to the acute indication. All analyses were conducted using SAS software, version 9.4 (SAS Institute Inc), and Stata SE, version 14 (StataCorp). OptumLabs data were analyzed from October 2018 to March 2019; MarketScan data were analyzed from January to April 2019.

## Results

Among 18 016 259 patients with private insurance and 11 453 392 Medicaid enrollees during the study period, 50.3% privately insured (eTable 1 in the Supplement) and 56.1% Medicaid-insured (eTable 2 in the Supplement) eligible patients were female and had a mean (95% CI) age of 42.7 (42.7-42.7) years (privately insured) and 20.4 (20.4-20.4) years (Medicaid), with a mean (95% CI) of 14 421 317 (14 326 784-14 515 840) privately insured patients and 9 742 711 (9 704 260-9 781 162) Medicaid enrollees per month. We identified 6 380 694 (35.4%) privately insured patients and 3 169 831 (27.7%) Medicaid enrollees with 1 or more visits that contained 1 or more pain-related diagnoses or surgical procedures from our indication list, including SCD and cancer. At least 1 opioid prescription during the study period was identified for 2 270 596 privately insured patients (35.6% of 6 380 694) and 1 126 508 Medicaid enrollees (35.5% of 3 169 831), and in total, this accounted for 87.7% of all 11 941 359 opioid prescriptions identified in 2017 OLDW data and 75.5% of all 7 157 341 opioid prescriptions in Medicaid data.

### Indications Associated With Nonsurgical Acute Pain

We identified 2 013 810 visits among patients with private insurance and 1 672 500 visits among Medicaid enrollees meeting our nonsurgical acute pain criteria. Overall, 13.4% privately insured patients and 15.6% Medicaid enrollees were linked with an opioid prescription. Ninety-seven percent of these linkages among privately insured patients and 98% among Medicaid enrollees involved a single prescription associated with a single visit.

Opioid prescribing rates associated with privately insured visits for nonsurgical acute pain ranged from 4.6% (acute migraines) to 44.8% (rib fractures) of visits (Table 1), slightly lower overall than for Medicaid-associated visits, which ranged from 6.6% (acute migraines) to 56.3% (rib fractures; Table 1). One notable exception was dental pain, for which patients with Medicaid were prescribed opioids in a lower percentage of visits (11.8%, Medicaid; 27.2%, privately insured), which may be partially attributable to the different patterns of dental claims included in each sample. Mean (95% CI) days’ supply varied, from 4.1 (4.1-4.2) days for dental pain (4.0 [4.0-4.0] days for Medicaid) to 11.8 (11.7-11.8) days for acute low back pain and 12.6 (12.3-12.8) days for acute migraine (9.9 [9.7-10.1] for acute migraine and 9.9 [9.8-10.0] for acute low back pain in Medicaid-covered visits). Mean daily opioid dosage was relatively constant across conditions in both data sets, approximately 30 MME/d, which is equivalent to 6 tablets containing a combination of 500 mg of acetaminophen and 5 mg of hydrocodone taken daily or to 4 tablets of oxycodone, 5 mg, taken daily.

**Table 1.** Opioid Prescribing Rates and Amounts Associated With Nonsurgical Acute Pain Indications Among Patients Not Receiving LTOT in the US, by Indication and Insurance Type, 2017^a^^,^^b^

Indication	Visits with Rx, No. (%) [95% CI]	No. of days’ supply		Daily dosage, MME	
		Mean (95% CI)	Median (IQR)	Mean (95% CI)	Median (IQR)
Abdominal pain					
Private insurance^c^	42 902 (11.5) [11.4-11.6]	5.9 (5.8-5.9)	4.0 (3.0-5.0)	31.4 (31.2-31.6)	27.0 (20.0-37.5)
Medicaid^d^	41 936 (13.0) [12.9-13.1]	4.8 (4.8-4.9)	3.0 (2.0-5.0)	30.2 (30.0-30.3)	25.0 (20.0-37.5)
Acute low back pain					
Private insurance	88 799 (13.7) [13.6-13.7]	11.8 (11.7-11.8)	7.0 (4.0-15.0)	27.7 (27.6-27.8)	21.4 (15.4-33.3)
Medicaid	61 217 (19.0) [18.9-19.2]	9.9 (9.8-10.0)	5.0 (3.0-15.0)	28.2 (28.0-28.4)	22.5 (16.9-33.3)
Acute migraine					
Private insurance	8221 (4.6) [4.5-4.7]	12.6 (12.3-12.8)	7.0 (4.0-20.0)	29.1 (28.6-29.7)	22.5 (15.0-36.0)
Medicaid	9797 (6.6) [6.5-6.7]	9.9 (9.7-10.1)	5.0 (3.0-15.0)	28.5 (28.1-28.9)	22.5 (16.7-33.8)
Dental pain					
Private insurance	19 127 (27.2) [26.9-27.6]	4.1 (4.1-4.2)	3.0 (2.0-5.0)	33.9 (33.6-34.1)	30.0 (21.4-45.0)
Medicaid	40 513 (11.8) [11.7-11.9]	4.0 (4.0-4.0)	3.0 (2.0-5.0)	28.0 (27.9-28.2)	25.0 (20.0-33.3)
Herpes zoster					
Private insurance	11 288 (15.5) [15.2-15.8]	8.4 (8.3-8.6)	5.0 (4.0-10.0)	27.3 (27.0-27.5)	22.5 (18.0-33.3)
Medicaid	3247 (26.1) [25.3-26.8]	6.3 (6.0-6.5)	4.0 (3.0-7.0)	28.3 (27.8-28.9)	25.0 (20.0-33.3)
Musculoskeletal sprains and strains					
Private insurance	69 434 (12.9) [12.8-13.0]	6.7 (6.6-6.7)	5.0 (3.0-7.0)	32.0 (31.9-32.2)	25.0 (20.0-37.5)
Medicaid	62 354 (14.8) [14.7-14.9]	5.1 (5.1-5.2)	3.0 (2.0-5.0)	28.0 (27.9-28.1)	25.0 (18.8-33.3)
Renal colic					
Private insurance	27 885 (20.1) [19.9-20.3]	5.2 (5.1-5.2)	4.0 (3.0-5.0)	35.5 (35.3-35.7)	30.0 (20.0-45.0)
Medicaid	16 618 (34.0) [33.6-34.4]	4.6 (4.6-4.7)	3.0 (2.0-5.0)	33.3 (33.0-33.6)	30.0 (20.0-40.0)
Rib fractures					
Private insurance	9455 (44.8) [44.1-45.4]	6.9 (6.7-7.0)	5.0 (3.0-8.0)	32.9 (32.5-33.3)	30.0 (20.0-40.0)
Medicaid	4484 (56.3) [55.2-57.4]	5.6 (5.5-5.8)	4.0 (3.0-6.0)	32.5 (31.9-33.1)	30.0 (20.0-37.5)

Abbreviations: IQR, interquartile range; LTOT, long-term opioid therapy; MME, morphine milligram equivalents^16^; Rx, prescriptions.

^a^ Reported outcome data (prescribing rate, MME, and days’ supply) reflect Rx supplied for a specific procedure or visit, meaning that the prescribing rate is anchored to visits or procedures.

^b^ Patients whose Rx do not meet LTOT criteria.

^c^ Data from the OptumLabs Data Warehouse, 2017.

^d^ Data from the MarketScan Multi-State Medicaid Database, from quarter 4 2016 to quarter 3 2017.

### Indications Associated With Chronic Pain

We identified 1 474 731 unique privately insured patients (eTable 1 in the Supplement) and 513 131 Medicaid patients meeting our chronic pain criteria (eTable 2 in the Supplement), among whom 22.7% privately insured patients and 18.6% Medicaid beneficiaries had several concurrent chronic pain indications. Back pain (radicular and nonradicular) was the most common chronic pain indication, affecting 49.3% of the 1 474 731 privately insured patients with chronic pain (52.2% of Medicaid enrollees) and 75.4% of privately insured patients with multiple concurrent chronic pain indications (80.4% of Medicaid enrollees). Overall, more than 30% of these 1 474 731 privately insured patients and almost 50% of these 513 131 Medicaid-covered patients had 1 or more opioid prescriptions linked to their chronic pain indication in the 3 months following the index diagnosis during the study period. Opioid prescribing rates and amounts differed markedly depending on chronic pain indication and opioid use history (Table 2).

**Table 2.** Opioid Prescribing Rates and Amounts Associated With Chronic Noncancer Pain Indications Among Patients in the US, by Indication, Insurance Type, and History of Opioid Use, 2017^a^

Indication	Patients with Rx, No. (%) [95% CI]	No. of Rx		No. of days’ supply		Daily dosage, MME	
		Mean (95% CI)	Median (IQR)	Mean (95% CI)	Median (IQR)	Mean (95% CI)	Median (IQR)
**Chronic nonradicular back pain**							
Privately insured^b^							
No LTOT^**c**^	113 112 (19.8) [19.7-19.9]	1.7 (1.7-1.7)	1 (1-2)	29.8 (29.6-30.0)	24 (8-35)	29.5 (29.4-29.7)	22.5 (15.0-37.5)
LTOT	103 270 (87.7) [87.5-87.8]	2.9 (2.9-2.9)	2 (1-4)	80.2 (79.9-80.6)	60 (30-107)	56.2 (55.8-56.5)	40.0 (27.5-62.0)
Medicaid^d^							
No LTOT	60 580 (32.6) [32.4-32.8]	1.9 (1.9-1.9)	1 (1-2)	30.7 (30.4-31.0)	20 (5-44)	28.6 (28.4-28.8)	22.5 (16.7-33.6)
LTOT	61 751 (90.4) [90.1-90.6]	2.8 (2.8-2.8)	2 (1-4)	74.0 (73.6-74.3)	60 (30-90)	47.7 (47.0-48.3)	37.5 (22.5-60.0)
**Chronic radicular back pain**							
Privately insured							
No LTOT	34 582 (28.3) [28.0-28.5]	1.8 (1.8-1.8)	1 (1-2)	34.9 (34.6-35.3)	30 (12-49)	30.2 (29.9-30.4)	22.5 (15.0-37.5)
LTOT	40 511 (87.5) [87.2-87.8]	2.9 (2.8-2.9)	2 (1-4)	80.5 (79.9-81.0)	60 (30-110)	58.5 (58.0-59.0)	45.0 (30.0-67.5)
Medicaid							
No LTOT	15 727 (44.0) [43.5-44.5]	2.0 (2.0-2.0)	2 (1-3)	36.6 (36.0-37.1)	30 (10-60)	28.7 (28.4-29.1)	22.5 (16.6-33.8)
LTOT	20 808 (88.2) [87.8-88.7]	2.7 (2.6-2.7)	2 (1-3)	71.8 (71.2-72.5)	60 (30-90)	47.5 (46.9-48.0)	37.5 (22.5-60.0)
**Chronic neck pain**							
Privately insured							
No LTOT	33 233 (15.8) [15.7-16.0]	1.7 (1.7-1.7)	1 (1-2)	29.9 (29.6-30.2)	23 (8-35)	30.6 (30.3-30.8)	25.0 (16.1-37.5)
LTOT	35 645 (87.3) [87.0-87.6]	2.9 (2.9-2.9)	2 (1-4)	81.3 (80.8-81.9)	60 (30-112)	59.4 (58.9-60.0)	44.4 (30.0-69.4)
Medicaid							
No LTOT	17 103 (31.1) [30.7-31.5]	1.9 (1.9-1.9)	1 (1-2)	32.3 (31.8-32.8)	24 (6-46)	28.8 (28.5-29.1)	22.5 (16.7-33.8)
LTOT	20 435 (88.4) [88.0-88.8]	2.7 (2.6-2.7)	2 (1-3)	71.1 (70.5-71.8)	60 (30-90)	48.4 (47.9-49.0)	40.0 (22.5-60.0)
**Fibromyalgia**							
Privately insured							
No LTOT	6489 (23.5) [23.0-24.0]	1.5 (1.5-1.5)	1 (1-2)	33.5 (32.8-34.2)	30 (15-35)	29.5 (28.8-30.1)	22.0 (15.0-36.0)
LTOT	9714 (77.9) [77.2-78.6]	2.4 (2.3-2.4)	2 (1-3)	67.2 (66.2-68.2)	60 (30-90)	56.3 (55.3-57.4)	40.0 (24.0-63.3)
Medicaid							
No LTOT	4239 (28.9) [28.2-29.7]	1.7 (1.7-1.7)	1 (1-2)	30.1 (29.2-31.0)	28 (7-35)	27.7 (27.1-28.3)	22.5 (15.0-33.3)
LTOT	5877 (81.7) [80.8-82.6]	2.3 (2.2-2.3)	2 (1-3)	61.8 (60.6-63.0)	58 (30-84)	44.2 (43.2-45.2)	30.0 (20.0-52.5)
**Inflammatory joint disorder**							
Privately insured							
No LTOT	90 261 (19.6) [19.5-19.7]	1.5 (1.5-1.5)	1 (1-2)	22.8 (22.7-23.0)	15 (7-30)	31.7 (31.6-31.9)	25.0 (17.5-40.0)
LTOT	56 676 (82.1) [81.8-82.4]	2.5 (2.5-2.5)	2 (1-3)	68.5 (68.1-68.9)	60 (30-90)	53.5 (53.1-53.9)	40.0 (24.6-60.0)
Medicaid							
No LTOT	46 566 (29.6) [29.4-29.8]	1.8 (1.8-1.8)	1 (1-2)	23.1 (22.9-23.4)	13 (5-30)	29.0 (28.8-29.2)	23.4 (17.5-33.8)
LTOT	34 096) (85.3) [84.9-85.6]	2.5 (2.4-2.5)	2 (1-3)	63.3 (62.8-63.7)	60 (30-90)	45.6 (45.1-46.1)	33.8 (22.5-58.1)
**Irritable bowel syndrome**							
Privately insured							
No LTOT	1524 (6.5) [6.2-6.8]	1.2 (1.2-1.3)	1 (1-1)	21.0 (20.0-21.9)	15 (6-30)	29.3 (28.1-30.4)	22.5 (15.0-37.5)
LTOT	1090 (63.4) [61.1-65.6]	1.8 (1.7-1.8)	1 (1-2)	48.1 (46.2-50.1)	30 (30-60)	48.3 (45.4-51.1)	34.5 (20.0-60.0)
Medicaid							
No LTOT	948 (13.4) [12.6-14.2]	1.4 (1.3-1.5)	1 (1-2)	16.8 (15.6-18.0)	10 (4-30)	29.1 (27.8-30.4)	22.5 (16.7-33.8)
LTOT	639 (66.4) [63.4-69.3]	1.7 (1.6-1.8)	1 (1-2)	44.2 (42.1-46.4)	30 (30-60)	40.4 (37.6-43.1)	30.0 (20.0-45.0)
**Nonmigraine headaches**							
Privately insured							
No LTOT	7826 (11.6) [11.4-11.9]	1.4 (1.4-1.4)	1 (1-1)	18.5 (18.0-18.9)	10 (5-30)	28.7 (28.2-29.2)	22.5 (16.3-33.8)
LTOT	5434 (75.0) [74.0-76.0]	2.1 (2.1-2.2)	2 (1-3)	56.4 (55.2-57.5)	35 (30-60)	52.6 (51.2-53.9)	40.0 (22.5-60.0)
Medicaid							
No LTOT	9255 (13.9) [13.6-14.1]	1.5 (1.4-1.5)	1 (1-2)	13.5 (13.1-13.8)	6 (3-16)	28.1 (27.7-28.5)	24.0 (18.0-33.3)
LTOT	4301 (74.1) [73.0-75.2]	1.9 (1.9-1.9)	1 (1-2)	45.8 (44.8-46.8)	30 (30-60)	43.9 (42.7-45.1)	30.0 (20.0-55.0)
**Osteoarthritis or joint cartilage conditions**							
Privately insured							
No LTOT	65 305 (18.8) [18.6-18.9]	1.4 (1.4-1.4)	1 (1-2)	23.6 (23.4-23.8)	17 (8-30)	31.6 (31.4-31.8)	25.0 (15.0-40.0)
LTOT	43 065 (77.6) [77.2-77.9]	2.2 (2.2-2.2)	2 (1-3)	60.1 (59.7-60.5)	53 (30-84)	49.1 (48.6-49.5)	37.5 (21.6-60.0)
Medicaid							
No LTOT	18 784 (33.2) [32.9-33.6]	1.7 (1.7-1.8)	1 (1-2)	27.1 (26.7-27.5)	20 (7-30)	30.2 (29.9-30.6)	23.4 (15.8-37.5)
LTOT	19 345 (83.1) [82.6-83.6]	2.3 (2.3-2.4)	2 (1-3)	60.9 (60.3-61.5)	56 (30-90)	44.5 (43.9-45.1)	33.8 (22.5-54.0)
**Periarticular or soft-tissue disorders**							
Privately insured							
No LTOT	27 773 (16.4) [16.2-16.6]	1.4 (1.4-1.4)	1 (1-2)	17.4 (17.2-17.6)	10 (5-26)	37.8 (37.5-38.1)	30.0 (20.0-50.0)
LTOT	12 683 (74.9) [74.3-75.6]	2.2 (2.2-2.2)	2 (1-3)	59.5 (58.7-60.2)	50 (30-75)	51.8 (51.0-52.7)	40.0 (22.5-60.0)
Medicaid							
No LTOT	9785 (28.3) [27.8-28.8]	1.7 (1.7-1.7)	1 (1-2)	19.9 (19.5-20.3)	12 (5-30)	32.4 (32.0-32.8)	27.8 (18.8-40.0)
LTOT	6254 (78.0) [77.1-78.9]	2.1 (2.0-2.1)	2 (1-3)	51.3 (50.5-52.2)	33 (30-60)	43.4 (42.3-44.4)	31.3 (21.3-52.5)

Abbreviations: IQR, interquartile range; LTOT, long-term opioid therapy; MME, morphine milligram equivalents^16^; Rx, prescriptions.

^a^ Reported outcome data (prescribing rate, MME, days’ supply, and number of Rx) are anchored to patients, reflecting all Rx supplied to a patient for visits related to that indication during the 3 months following the index diagnosis.

^b^ Data from the OptumLabs Data Warehouse, 2017.

^c^ Patients whose Rx do not meet LTOT criteria.

^d^ Data from the MarketScan Multi-State Medicaid Database, from quarter 4 2016 to quarter 3 2017.

#### Patients Not Receiving LTOT

We categorized 87.4% of 1 474 731 privately insured patients and 80.0% of 513 131 Medicaid-insured patients with chronic pain as not receiving LTOT based on our criteria. Among privately insured patients not receiving LTOT, opioid prescribing rates ranged from 6.5% (irritable bowel syndrome) to 28.3% (chronic radicular back pain), overall lower than rates for patients with Medicaid, which ranged from 13.4% (irritable bowel syndrome) to 44.0% (chronic radicular back pain). Mean days’ supply ranged from 17.4 to 34.9 days (privately insured) and 13.5 to 36.6 days (Medicaid); patients with back pain, neck pain, and fibromyalgia received longer days’ supplies (typically approaching or exceeding 30 days) than other patients not receiving LTOT with chronic pain across both patient samples. An approximate mean dosage of 30 MME/d was associated with most conditions.

#### Patients Already Receiving LTOT

We categorized 12.6% of privately insured patients and 20.0% of Medicaid enrollees with chronic pain as already receiving LTOT prior to the study index diagnosis. Among them, 60.4% (privately insured) and 61.2% (Medicaid) had the same chronic pain indication in the lookback period prior to the study index diagnosis, suggesting that for the majority of patients already receiving LTOT, it is likely that their LTOT was for this same preexisting chronic pain indication. Patients already receiving LTOT were highly likely to continue to receive opioids coincident with a visit for a chronic pain diagnosis, ranging from 63.4% of privately insured patients (66.4% for Medicaid insured) with irritable bowel syndrome to 87.7% of patients (90.4% for Medicaid insured) with chronic nonradicular back pain. Most patients already prescribed LTOT with 1 or more prescriptions linked to a visit for a chronic pain diagnosis received sufficient opioids for at least half the days of the 3 months following the visit across both patient populations. For privately insured patients already prescribed LTOT, mean daily dosage exceeded 50 MME per day for all conditions except irritable bowel syndrome (48.3; 95% CI, 45.4-51.1 MME) and osteoarthritis or joint cartilage conditions (49.1; 95% CI, 48.6-49.5 MME), in contrast to Medicaid enrollees who received fewer than 50 MME per day for the chronic conditions under study.

### Indications Associated With Postsurgical Pain

In total, we identified 385 254 surgical procedures among privately insured patients and 285 996 among Medicaid enrollees. Overall, opioids were prescribed at hospital discharge for 66% of these procedures for the privately insured patients and 55% for Medicaid enrollees (Table 3). The lowest hospital discharge prescribing rates among patients not receiving LTOT occurred after vaginal delivery (private insurance: 23.6%; 95% CI, 23.3%-23.9% vs Medicaid: 30.7%; 95% CI, 30.4%-30.9%), open colectomy (private insurance: 34.8%; 95% CI, 33.2%-36.4% vs Medicaid: 35.6%; 95% CI, 32.9%-38.4%), coronary artery bypass surgery (private insurance: 34.8%; 95% CI, 33.7%-35.8% vs Medicaid: 39.8%; 95% CI, 37.0%-42.6%), and tonsillectomy (private insurance: 44.2%; 95% CI, 43.3%-45.0% vs Medicaid, 35.9%; 95% CI, 35.4%-36.4%) (Table 3). Among privately insured patients, arthroscopic rotator cuff repair (93.0% of procedures), arthroscopic knee surgery (92.9%), lumbar decompression (86.0%), and laparoscopic abdominal solid organ resection (85.9%) had the highest rates, whereas among Medicaid enrollees, the highest rates were observed for arthroscopic rotator cuff repair (94.4%), laparoscopic abdominal solid organ resection (92.4%), arthroscopic knee surgery (92.1%), and laparoscopic cholecystectomy (87.6%). Mean days’ supply of opioids for postsurgical pain among patients not receiving LTOT ranged from 4.1 days (95% CI, 4.1-4.1 days) for vaginal delivery to 9.5 days (95% CI, 9.4-9.7 days) for combined spinal fusion and lumbar decompression in the privately insured population, and 4.2 (95% CI, 4.2-4.2 days) for vaginal delivery to 9.1 (95% CI, 8.9-9.2 days) for spinal fusion in the Medicaid population. Mean (95% CI) daily dosage across all indications among privately insured patients ranged from 37.4 (37.0-37.9) MME for lumpectomies or partial mastectomies to 63.5 (62.5-64.4) MME for combined spinal fusion and lumbar decompression; among Medicaid-insured patients, the range was 27.3 (27.0-27.7) MME for tonsillectomies to 62.9 (60.4-65.4) MME for combined spinal fusion and lumbar decompression.

**Table 3.** Opioid Prescribing Rates and Amounts for Postsurgical Pain Management Among Patients Not Receiving Long-term Opioid Therapy in the US, by Indication and Insurance Type, 2017^a^^,^^b^

Procedure	Private insurance^c^					Medicaid^d^				
	Procedures with Rx, No. (%) [95% CI]	No. of days’ supply		Daily dosage, MME		Procedures with Rx, No. (%) [95% CI]	No. of days’ supply		Daily dosage, MME	
		Mean (95% CI)	Median (IQR)	Mean (95% CI)	Median (IQR)		Mean (95% CI)	Median (IQR)	Mean (95% CI)	Median (IQR)
Vaginal delivery	14 166 (23.6) [23.3-23.9]	4.1 (4.1-4.1)	4.0 (3.0-5.0)	39.0 (38.8-39.3)	37.5 (27.0-50.0)	35 208 (30.7) [30.4-30.9]	4.2 (4.2-4.2)	4.0 (3.0-5.0)	38.2 (38.0-38.4)	33.3 (25.0-45.0)
Total knee arthroplasty	27 528 (79.5) [79.1-79.9]	8.5 (8.5-8.6)	8.0 (5.0-10.0)	62.0 (61.6-62.3)	57.7 (37.5-85.7)	2753 (85.7) [84.5-86.9]	8.3 (8.2-8.5)	7.0 (5.0-10.0)	61.1 (60.0-62.3)	55.6 (30.9-85.7)
Sinus surgery	21 661 (63.5) [63.0-64.0]	4.9 (4.8-4.9)	4.0 (3.0-5.0)	42.2 (41.9-42.6)	40.0 (30.0-50.0)	5905 (59.8) [58.9-60.8]	5.3 (5.2-5.4)	5.0 (3.0-7.0)	39.9 (39.2-40.6)	37.5 (25.0-50.0)
Cholecystectomy, laparoscopic	23 664 (80.2) [79.7-80.6]	4.7 (4.7-4.8)	4.0 (3.0-5.0)	42.1 (41.8-42.3)	37.5 (28.6-50.0)	14 081 (87.6) [87.1-88.2]	4.8 (4.7-4.8)	4.0 (3.0-5.0)	42.3 (42.0-42.6)	37.5 (25.0-50.0)
Cesarean section	24 247 (78.0) [77.6-78.5]	4.9 (4.9-5.0)	5.0 (3.0-5.0)	48.5 (48.2-48.7)	45.0 (31.5-60.0)	42 560 (83.3) [83.0-83.6]	5.1 (5.1-5.1)	5.0 (4.0-6.0)	49.3 (49.1-49.5)	45.0 (32.1-60.0)
Total hip arthroplasty	14 375 (76.4) [75.8-77.0]	8.4 (8.3-8.4)	7.0 (5.0-10.0)	57.6 (57.1-58.0)	50.0 (31.3-80.0)	1745 (83.4) [81.8-85.0]	8.4 (8.2-8.6)	7.0 (5.0-10.0)	61.6 (60.0-63.3)	56.3 (30.8-85.7)
Lumpectomy or partial mastectomy	6355 (69.8) [68.9-70.8]	4.2 (4.2-4.3)	4.0 (3.0-5.0)	37.4 (37.0-37.9)	33.3 (25.0-50.0)	2073 (81.0) [79.5-82.5]	4.7 (4.6-4.8)	5.0 (3.0-5.0)	37.3 (36.5-38.1)	32.5 (25.0-45.0)
Combined spinal fusion and lumbar decompression surgery	10 620 (75.5) [74.8-76.2]	9.5 (9.4-9.7)	8.0 (5.0-10.0)	63.5 (62.5-64.4)	57.1 (35.0-85.7)	1796 (74.1) [72.3-75.8]	8.9 (8.6-9.2)	8.0 (5.0-10.0)	62.9 (60.4-65.4)	56.3 (33.3-85.7)
Lumbar decompression	11 844 (86.0) [85.4-86.6]	8.1 (8.0-8.1)	7.0 (5.0-10.0)	53.9 (53.5-54.4)	45.0 (30.0-67.5)	1987 (81.9) [80.4-83.5]	8.1 (7.9-8.3)	7.0 (5.0-10.0)	50.1 (49.0-51.3)	45.0 (30.0-64.3)
Arthroscopic rotator cuff repair	12 538 (93.0) [92.6-93.5]	6.6 (6.6-6.7)	5.0 (5.0-8.0)	62.6 (62.1-63.1)	60.0 (40.0-84.4)	1837 (94.4) [93.4-95.4]	7.3 (7.1-7.4)	7.0 (5.0-10.0)	56.4 (55.3-57.6)	50.0 (30.0-75.0)
Spinal fusion	9206 (81.3) [80.6-82.0]	9.0 (8.9-9.1)	8.0 (5.0-10.0)	58.4 (57.7-59.0)	50.0 (31.3-75.0)	3136 (78.5) [77.2-79.8]	9.1 (8.9-9.2)	8.0 (5.0-10.0)	54.7 (53.7-55.8)	45.0 (30.0-75.0)
Tonsillectomy	6229 (44.2) [43.3-45.0]	7.2 (7.1-7.4)	7.0 (5.0-9.0)	42.2 (41.5-42.9)	34.6 (19.3-59.1)	12 800 (35.9) [35.4-36.4]	7.4 (7.3-7.5)	7.0 (5.0-10.0)	27.3 (27.0-27.7)	21.4 (12.5-35.0)
Abdominal solid organ resection, laparoscopic	10 197 (85.9) [85.3-86.5]	5.0 (4.9-5.0)	5.0 (3.0-6.0)	46.2 (45.7-46.6)	45.0 (30.0-56.3)	4531 (92.4) [91.7-93.2]	5.1 (5.0-5.2)	5.0 (3.0-7.0)	46.7 (46.0-47.3)	45.0 (30.0-60.0)
Appendectomy, laparoscopic	8649 (69.0) [68.2-69.8]	4.6 (4.5-4.6)	4.0 (3.0-5.0)	42.4 (42.0-42.8)	37.5 (30.0-50.0)	5605 (64.9) [63.8-65.9]	4.5 (4.4-4.5)	4.0 (3.0-5.0)	36.9 (36.3-37.4)	30.0 (20.4-45.0)
Inguinal hernia repair, open	8250 (78.0) [77.2-78.8]	4.9 (4.8-4.9)	5.0 (3.0-5.0)	43.0 (42.6-43.5)	40.0 (30.0-50.0)	2580 (61.9) [60.5-63.4]	5.0 (4.9-5.1)	5.0 (3.0-6.0)	34.6 (33.7-35.5)	30.0 (15.0-46.9)
Excisional biopsy	2148 (48.5) [47.0-49.9]	5.1 (4.9-5.2)	5.0 (3.0-6.0)	41.1 (39.9-42.3)	35.0 (27.0-50.0)	1051 (49.5) [47.4-51.7]	5.1 (4.9-5.3)	5.0 (3.0-7.0)	37.9 (36.6-39.3)	30.0 (20.8-45.0)
Coronary artery bypass	2835 (34.8) [33.7-35.8]	7.2 (7.0-7.3)	6.0 (5.0-9.0)	44.0 (43.2-44.9)	40.0 (30.0-56.3)	463 (39.8) [37.0-42.6]	7.1 (6.7-7.5)	7.0 (5.0-9.0)	46.8 (44.1-49.6)	42.9 (28.6-57.1)
Inguinal hernia repair, laparoscopic	6614 (83.0) [82.2-83.9]	4.8 (4.7-4.8)	4.0 (3.0-5.0)	45.3 (44.8-45.8)	45.0 (30.0-56.3)	1040 (79.1) [77.0-81.3]	5.2 (5.0-5.3)	5.0 (3.0-7.0)	42.9 (41.5-44.3)	40.0 (25.0-56.3)
Simple mastectomy	2096 (83.3) [81.9-84.8]	5.5 (5.3-5.6)	5.0 (4.0-7.0)	51.1 (49.8-52.4)	45.0 (30.0-64.3)	338 (87.3) [84.0-90.7]	5.6 (5.2-6.0)	5.0 (4.0-7.0)	48.6 (45.6-51.7)	45.0 (30.0-60.0)
Arthroscopic knee surgery	6887 (92.9) [92.3-93.5]	6.2 (6.2-6.3)	5.0 (4.0-7.0)	60.9 (60.3-61.6)	56.3 (40.0-75.0)	3021 (92.1) [91.2-93.0]	6.8 (6.7-6.9)	6.0 (5.0-8.0)	54.0 (53.1-55.0)	46.9 (30.0-75.0)
Colectomy, laparoscopic	2316 (60.4) [58.9-62.0]	5.3 (5.2-5.4)	5.0 (3.0-7.0)	45.8 (44.9-46.8)	43.8 (30.0-56.3)	403 (64.6) [60.8-68.3]	5.5 (5.2-5.8)	5.0 (4.0-7.0)	50.4 (47.6-53.2)	45.0 (30.0-60.0)
Parathyroid or thyroid surgery	3057 (71.1) [69.8-72.5]	4.8 (4.7-4.8)	4.0 (3.0-5.0)	41.7 (41.0-42.4)	37.5 (30.0-50.0)	1267 (81.8) [79.9-83.7]	5.2 (5.0-5.3)	5.0 (3.0-7.0)	43.1 (41.9-44.3)	37.5 (28.6-50.0)
Cholecystectomy, open	512 (50.5) [47.4-53.6]	5.4 (5.1-5.6)	5.0 (3.0-6.0)	41.3 (39.5-43.0)	37.5 (28.1-50.0)	234 (58.9) [54.1-63.8]	5.3 (4.9-5.6)	5.0 (3.0-7.0)	45.2 (42.4-48.1)	38.8 (28.1-60.0)
Colectomy, open	1196 (34.8) [33.2-36.4]	5.7 (5.5-5.9)	5.0 (4.0-7.0)	43.5 (42.2-44.9)	37.5 (28.1-50.0)	422 (35.6) [32.9-38.4]	5.5 (5.3-5.7)	5.0 (3.0-7.0)	48.9 (45.8-52.1)	41.7 (28.6-60.0)

Abbreviations: IQR, interquartile range; MME, morphine milligram equivalents^16^; Rx, prescriptions.

^a^ Reported outcome data (prescribing rate, MME, and days’ supply) reflect Rx supplied for a specific procedure or visit, meaning that the prescribing rate is anchored to visits or procedures.

^b^ Patients whose Rx do not meet LTOT criteria.

^c^ Data from the OptumLabs Data Warehouse, 2017.

^d^ Data from the MarketScan Multi-State Medicaid Database, from quarter 4 2016 to quarter 3 2017.

Across both patient populations, patients already receiving LTOT who underwent surgery nearly always were prescribed opioids to treat postsurgical pain. Also, postsurgical mean days’ supply and mean daily dosage was nearly universally higher among patients already receiving LTOT across both patient populations (eTable 3 in the Supplement).

### Sickle Cell Disease

Almost half of privately insured patients (42.6%) and Medicaid-enrolled patients (44.9%) with SCD received an opioid prescription overall (Table 4). However, there were marked differences by age. More than twice as many children with SCD covered under Medicaid received an opioid prescription (29.0%) compared with children with SCD and private insurance (12.2%). A higher percentage of nonelderly adults with SCD and Medicaid received opioids, with a mean (95% CI) days’ supply nearly twice as high (117.3 [112.1-122.6] days) as that prescribed for those with private insurance (59.2 [53.8-64.6] days).

**Table 4.** Opioid Prescribing Rates and Amounts Among Patients With Sickle Cell Disease in the US, by Age Group and Insurance Type, 2017^a^

Insurance type	Patients with Rx, No. (%) [95% CI]	No. of Rx		No. of days’ supply		Daily dosage, MME	
		Mean (95% CI)	Median (IQR)	Mean (95% CI)	Median (IQR)	Mean (95% CI)	Median (IQR)
At 0-18 y							
Private^b^	34 (12.2) [8.4-16.1]	1.8 (1.3-2.3)	1 (1-2)	15.7 (6.0-25.5)	6 (3-12)	34.3 (28.6-40.0)	30.2 (24.6-46.5)
Medicaid^c^	1279 (29.0) [27.6-30.3]	3.4 (3.2-3.5)	2 (2-4)	24.4 (22.4-26.4)	14 (10-24)	31.9 (30.8-33.1)	30 (18-45)
At 19-64 y							
Private	479 (52.5) [49.2-55.7]	3.2 (3.0-3.4)	2 (1-4)	59.2 (53.8-64.6)	40 (15-87)	85.1 (76.7-93.5)	58.1 (35.9-106.7)
Medicaid	1829 (72.9) [71.2-74.7]	7.5 (7.2-7.8)	6 (4-10)	117.3 (112.1-122.6)	80 (30-180)	88.8 (85.3-92.4)	60 (40-108)
At ≥65 y							
Private	45 (37.5) [28.8-46.2]	2.5 (2.0-3.0)	2 (1-3)	54.1 (39.8-68.3)	38 (18-76)	54.1 (40.6-67.5)	40.0 (21.4-72.6)
Medicaid	NA	NA	NA	NA	NA	NA	NA
Overall							
Private	558 (42.6) [39.9-45.2]	3.0 (2.8-3.3)	2 (1-4)	56.1 (51.3-61.0)	34 (12-82)	79.5 (72.1-86.9)	52.5 (30.7-96.0)
Medicaid	3108 (44.9) [43.8-46.1]	5.8 (5.6-6.0)	4 (2-8)	79.1 (75.5-82.7)	32 (14-110)	65.6 (63.2-67.9)	45 (28-75)

Abbreviations: IQR, interquartile range; MME, morphine milligram equivalents^16^; Rx, prescriptions.

^a^ Reported outcome data (prescribing rate, MME, days’ supply, and number of Rx) are anchored to patients, reflecting all Rx supplied to a patient for visits related to that indication during the 3 months following the index diagnosis.

^b^ Data from the OptumLabs Data Warehouse, 2017.

^c^ Data from the MarketScan Multi-State Medicaid Database, from quarter 4 2016 to quarter 3 2017.

### Cancer

A third of privately insured patients with cancer received an opioid prescription compared with more than half (56.6%) of patients with Medicaid. Patients with Medicaid received a higher days’ supply and dosage compared with privately insured patients (Medicaid: 115.2 [95% CI, 112.9-117.5] days; 61.1 [95% CI, 59.9-62.4] MME/d; privately insured: 34.2 [95% CI, 33.8-34.6] days; 46.2 [95% CI, 45.8-46.5] MME/d) (Table 5).

**Table 5.** Opioid Prescribing Rates and Amounts Among Patients With Cancer in the US, by Age Group and Insurance Type, 2017^a^

Insurance type	Patients with Rx, No. (%) [95% CI]	No. of Rx		No. of days’ supply		Daily dosage, MME	
		Mean (95% CI)	Median (IQR)	Mean (95% CI)	Median (IQR)	Mean (95% CI)	Median (IQR)
At 0-18 y							
Private^b^	244 (23.0) [20.4-25.5]	1.8 (1.6-2.0)	1 (1-2)	13.4 (10.0-16.9)	7 (4-12)	27.7 (25.3-30.1)	23.9 (14.1-39.3)
Medicaid^c^	489 (25.5) [23.5-27.4]	3.9 (3.6-4.2)	2 (2-4)	31.0 (26.3-35.6)	16 (10-30)	28.2 (26.1-30.4)	23 (12-38)
At 19-64 y							
Private	18 588 (40.6) [40.1-41.0]	2.4 (2.4-2.5)	2 (1-3)	37.5 (36.8-38.2)	15 (5-50)	52.6 (51.9-53.2)	42.8 (30.0-60.0)
Medicaid	8854 (60.9) [60.1-61.6]	6.9 (6.8-7.0)	6 (2-10)	120.9 (118.5-123.4)	86 (30-180)	63.5 (62.2-64.8)	45 (30-75)
At ≥65 y							
Private	27 551 (27.7) [27.4-27.9]	2.0 (2.0-2.0)	1 (1-2)	32.2 (31.8-32.7)	15 (5-41)	42.0 (41.6-42.4)	33.5 (22.5-50.0)
Medicaid	323 (54.4) [50.4-58.4]	5.4 (4.9-5.9)	4 (2-6)	86.1 (75.8-96.4)	60 (18-124)	45.3 (41.0-49.5)	35 (25-51)
Overall							
Private	46 383 (31.7) [31.4-31.9]	2.2 (2.1-2.2)	1 (1-3)	34.2 (33.8-34.6)	15 (5-45)	46.2 (45.8-46.5)	37.5 (25.0-55.0)
Medicaid	9666 (56.6) [55.9-57.4]	6.7 (6.6-6.8)	6 (2-8)	115.2 (112.9-117.5)	74 (24-180)	61.1 (59.9-62.4)	45 (30-71)

Abbreviations: IQR, interquartile range; MME, morphine milligram equivalents^16^; Rx, prescriptions.

^a^ Reported outcome data (prescribing rate, MME, days’ supply, and number of Rx) are anchored to patients, reflecting all Rx supplied to a patient for visits related to that indication during the 3 months following the index diagnosis.

^b^ Data from the OptumLabs Data Warehouse, 2017.

^c^ Data from the MarketScan Multi-State Medicaid Database, from quarter 4 2016 to quarter 3 2017.

### Opioid Prescribing Variation by Age Group

Opioids were prescribed for fewer patients and visits, and in lower amounts, for children aged 18 years or younger compared with adults for most indications (Table 4 and Table 5; eTables 4-9 in the Supplement). Compared with adults, children prescribed opioids received shorter durations for most indications; lower dosages for SCD, postsurgical pain, and cancer; and similar dosages for nonsurgical acute pain. There were insufficient data to report on pediatric patients with private insurance prescribed opioids for chronic pain, but among children with Medicaid coverage, a lower percentage received opioids for chronic pain compared with adults (eg, 4.4% of children vs 37.4% of nonelderly adults among Medicaid enrollees not receiving LTOT with nonradicular back pain).

Opioids were prescribed in a lower percentage of visits for adults aged 65 years or older for dental pain, renal colic, most surgical procedures, and cancer compared with other adults, but prescribing rates were similar to those for other adults for most other nonsurgical acute and chronic pain indications (Table 4 and Table 5; eTables 4-9 in the Supplement). Days’ supply and dosages were similar for adults aged 19 to 64 years and those aged 65 years or older, except that adults younger than 65 years who were also receiving LTOT, who underwent surgery, or had SCD or cancer received higher mean daily dosages than older adults in these categories.

## Discussion

In our analysis, we linked opioid prescriptions to 13% to 16% of nonsurgical acute pain visits, 30% to 50% of patients with chronic pain indications, 55% to 66% of surgical procedures, 43% to 45% of patients with SCD, and 32% to 57% of patients with cancer, with rates, days’ supply, and daily dosage varying widely across indications. For many indications, opioid prescribing rates, days’ supply, and dosage did not align with evidence-based guidelines and practice-informed recommendations. For example, published guidance recommends nonopioid treatment of fibromyalgia,^17,18^ chronic^17,19,20^ and acute back pain,^17,19,20^ musculoskeletal strains or sprains,^21^ and dental pain^22,23^ with no more than 3 to 7 days of opioids when needed for acute pain.^6^ Opioid prescriptions were issued to patients for all of these indications across both study populations in this analysis. We found that across both patient populations, patients with fibromyalgia not already receiving LTOT were typically prescribed at least a full month’s supply of opioids; that 28.3% (privately insured) and 44.0% (Medicaid) of patients with chronic radicular back pain not already receiving LTOT were started on opioids; and that opioid prescriptions with mean treatment durations of 11.8 (privately insured) and 9.9 days (Medicaid) were issued for acute low back pain.

For many patients with chronic pain conditions receiving LTOT, daily dosages of opioids exceeded 50 MME, a threshold beyond which the risk for adverse events, including overdose, is increased.^6^ Previous analyses have found associations between longer durations of opioid therapy and higher opioid dosages,^24^ potentially reflecting increasing dosages as patients develop tolerance to pain-relieving effects of opioids. It is important for clinicians to increase long-term opioid dosages only when it is clear that benefits of increasing dosage will outweigh risks.^6^ Once patients are receiving high opioid dosages long term, it can be difficult to reverse course.^25^ Successful dosage reduction is likely to require strong collaboration between the patient and clinician, behavioral support, multimodal pain treatment, and time (often months to years) to taper slowly enough to minimize withdrawal symptoms.^6,26^

Postoperative opioid prescribing at hospital discharge exceeds published recommendations for nearly all surgical procedures included in this analysis. For example, the Michigan Surgical Quality Collaborative^27^ recommends up to 10 tablets of oxycodone, 5 mg, at discharge for opioid-naive patients after laparoscopic cholecystectomy, totaling 75 MME. One tablet taken every 4 hours would exhaust a 10-tablet supply in less than 2 days; we found 4.7 (privately insured) and 4.8 (Medicaid) days’ supply provided to patients not receiving LTOT. Numerous studies report that patients frequently take fewer opioids than prescribed.^11,28,29,30,31^ Therefore, larger amounts of prescribed opioids suggest excess opioids potentially accessible to others, increasing risks of misuse and overdose. To minimize risks of excess opioids while meeting individual pain control needs, 1 institution^32^ calculates postsurgical discharge opioid prescriptions based on inpatient opioid use on the day prior to discharge.

Among patients with cancer, about one-third in private insurance and about half in Medicaid were prescribed opioids. One study of outpatients with recurrent or metastatic cancer found that 62% reported moderate to severe pain,^33^ for which opioids are recommended.^34^ It is possible that many patients with cancer in our sample did not have pain or had pain that was managed effectively with nonopioid treatment. However, some patients might not have received pain management proportionate to their pain severity, as has been reported previously.^12^

Among patients with SCD, fewer than half with either insurance type were prescribed opioids. Suboptimal management of acute SCD pain has been reported^13,14^ and might have affected some patients. However, claims data cannot identify whether prescriptions were associated with acute sickle cell crisis or with chronic pain. Privately insured adults and those with Medicaid diagnosed as having SCD were much more likely than children to receive opioids, and adults received substantially higher opioid dosages and for longer durations than children did. This finding is consistent with previous studies^35^ and likely reflects increased incidence of SCD complications as individuals age. Other studies have found that adults and patients with Medicaid were less likely to fill hydroxyurea prescriptions or receive specialty care for SCD,^35^ suggesting that lack of access to care may be associated with complications such as vaso-occlusive crisis and with a need to treat complications with opioids. In addition, clinicians often do not offer multimodal pain management for SCD pain,^14^ and many adults with SCD use opioids for chronic pain as well as for acute pain crises,^14^ which may increase tolerance and opioid requirements when patients experience acute pain. Other research has found that opioid use among SCD patients has been substantial but stable across recent years (2008-2012), while use increased in the broader US population, and that opioid-related overdose deaths among individuals with SCD were substantially lower than among individuals with other diseases associated with pain.^35^ Because of the unique challenges in managing painful SCD complications, readers of the 2016 *CDC Guideline for Prescribing Opioids for Chronic Pain* were referred to other guidelines specific to SCD for more guidance.^36^ Despite this, there have been reports of inappropriate misapplication of CDC guideline recommendations to patients with SCD^14^ as well as with cancer pain,^37^ and the CDC has released statements emphasizing that the intended, stated scope of the guideline did not include pain in the setting of acute cancer or acute sickle cell crisis.^37^

Overall, opioid prescribing rates were higher among Medicaid than privately insured patients, but Medicaid patients were prescribed lower daily dosages and shorter durations. These findings merit closer examination; they may reflect greater use of drug utilization strategies by Medicaid in comparison with commercial payers, such as prior authorization and condition or referral requirements for use of nonopioid medications and nonpharmacologic therapies.^38^

### Limitations

Caution is needed in interpreting several aspects of this analysis. We relied on claims data and were unable to assess clinical details, such as pain severity or function, or to assess opioid prescriptions not covered by insurance. Other pain treatments that patients may have received, such as nonopioid medications or nonpharmacologic treatments, were not directly assessed. We did not assess uninsured patients, those with fee-for-service Medicare, or those whose prescriptions were covered under other means, including cash payments or other insurance plans. Variation in plans and inability to identify specific state Medicaid populations in the MMD data limit interpretation of prescribing rates in the context of opioid prescribing policies, such as prior authorization and duration limits. We used a linkage procedure to connect opioid prescriptions to the most likely indications at the patient or visit level; some prescriptions could have been misattributed to specific indications that were not the actual intent of the prescription or intended for an indication not included in our analysis. However, the patients identified using our list of pain indications accounted for the vast majority (87.7%) of total opioid prescriptions among privately insured patients in the OLDW data set in 2017. Dental procedures in OLDW were those covered by medical benefits and may not be representative of dental procedures for which medical claims are not billed. By contrast, the Medicaid data included direct claims from dentists, limiting direct comparisons between insurance plans. In the SCD analysis, we are unable to differentiate between LTOT for SCD and opioids prescribed to treat an acute vaso-occlusive crisis, and the small number of patients with SCD and private insurance may not represent the general SCD population. The amount of time that elapsed between the date of an inpatient surgical procedure and hospital discharge may affect the amount of pain medication prescribed for an individual patient at the point of discharge. Our outcome of interest was overall prescribing for specific indications, rather than prescribing by individual prescribers owing to data set limitations, which may affect estimates of precision. Lastly, prescribing rates do not reflect national estimates of overall opioid prescribing; rates are based on specific patient populations for specific indications, and geographic constraints of the data sources used in this analysis may limit national application of calculated rates.

## Conclusions

This comprehensive analysis of opioid prescribing across a broad variety of indications suggested that prescribing patterns for some indications were incongruent with existing evidence-based clinical guidelines. These results may reflect low clinician awareness of applicable guidelines or reluctance to adhere to guidance. Implementation guidance that emphasizes evidence-based recommendations has the potential to better align opioid prescribing practices with evidence on opioid benefits and risks and improve pain management and patient safety.

## Notes

Supplement. **eAppendix 1.** Supplementary Methods **eAppendix 2.** Indications Associated With Pain **eTable 1.** Summary Statistics of Privately Insured Population by Pain Indication, 2017 **eTable 2.** Summary Statistics of Medicaid Population by Pain Indication, Q4 2016 to Q3 2017 **eTable 3.** Opioid Prescribing Rates and Amounts for Postsurgical Pain Management Among Patients on Long-term Opioid Therapy in the United States, by Indication and Insurance Type, 2017 **eTable 4.** Opioid Prescribing Rates and Amounts for Nonsurgical Acute Pain Among Privately Insured Patients Not on Long-term Opioid Therapy in the United States, by Indication and Age Group, 2017 **eTable 5.** Opioid Prescribing Rates and Amounts for Chronic Noncancer Pain Among Privately Insured Patients in the United States, by Indication, History of Opioid Usage, and Age Group, 2017 **eTable 6.** Opioid Prescribing Rates and Amounts for Postsurgical Pain Management Among Privately Insured Patients Not on Long-term Opioid Therapy in the United States, by Indication and Age Group, 2017 **eTable 7.** Opioid Prescribing Rates and Amounts for Nonsurgical Acute Pain Among Medicaid Enrollees Not on Long-term Opioid Therapy in the United States, by Age Group, Q4 2016–Q3 2017 **eTable 8.** Opioid Prescribing Rates and Amounts for Chronic Noncancer Pain Among Medicaid Enrollees in the United States, by Indication, Timing of Chronic Pain Diagnosis, History of Opioid Usage, and Age Group, Q4 2016–Q3 2017 **eTable 9.** Opioid Prescribing Rates and Amounts for Postsurgical Pain Management Among Medicaid Enrollees Not on Long-term Opioid Therapy in the United States, by Indication and Age Group, Q4 2016–Q3 2017 **eReferences.**

## References

[1] Scholl L, Seth P, Kariisa M, Wilson N, Baldwin G Drug and opioid-involved overdose deaths—United States, 2013-2017. MMWR Morb Mortal Wkly Rep. 2018;67(5152):1419-1427. doi:10.15585/mmwr.mm675152e1 30605448 PMC6334822

[2] Substance Abuse and Mental Health Services Administration Key substance use and mental health indicators in the United States: results from the 2018 National Survey on Drug Use and Health. Accessed March 31, 2020. https://store.samhsa.gov/product/key-substance-use-and-mental-health-indicators-in-the-united-states-results-from-the-2018-national-survey-on-Drug-Use-and-Health/PEP19-5068

[3] Cicero TJ, Ellis MS, Kasper ZA Increased use of heroin as an initiating opioid of abuse. Addict Behav. 2017;74:63-66. Published online May 23, 2017. doi:10.1016/j.addbeh.2017.05.030 28582659

[4] Edlund MJ, Martin BC, Russo JE, DeVries A, Braden JB, Sullivan MD The role of opioid prescription in incident opioid abuse and dependence among individuals with chronic noncancer pain: the role of opioid prescription. Clin J Pain. 2014;30(7):557-564. 24281273 10.1097/AJP.0000000000000021 PMC4032801

[5] Bohnert ASB, Valenstein M, Bair MJ, Association between opioid prescribing patterns and opioid overdose-related deaths. JAMA. 2011;305(13):1315-1321. doi:10.1001/jama.2011.370 21467284

[6] Dowell D, Haegerich TM, Chou R CDC guideline for prescribing opioids for chronic pain—United States, 2016. MMWR Recomm Rep. 2016;65(1):1-49. doi:10.15585/mmwr.rr6501e1 26987082

[7] Department of Veteran Affairs and Department of Defense. The Opioid Therapy for Chronic Pain Workgroup. VA/DoD clinical practice guideline for opioid therapy for chronic pain. Accessed March 31, 2020. https://www.healthquality.va.gov/guidelines/Pain/cot/VADoDOTCPG022717.pdf

[8] Washington State Agency Medical Director’s Group Prescribing opioids for postoperative pain–supplemental guidance. Published July 2019. Accessed March 31, 2020. http://www.agencymeddirectors.wa.gov/Files/FinalSupBreeAMDGPostopPain091318wcover.pdf

[9] Shah A, Hayes CJ, Martin BC Factors influencing long-term opioid use among opioid naive patients: an examination of initial prescription characteristics and pain etiologies. J Pain. 2017;18(11):1374-1383. doi:10.1016/j.jpain.2017.06.010 28711636 PMC5660650

[10] Eid AI, DePesa C, Nordestgaard AT, Variation of opioid prescribing patterns among patients undergoing similar surgery on the same acute care surgery service of the same institution: time for standardization? Surgery. 2018;164(5):926-930. doi:10.1016/j.surg.2018.05.047 30049481

[11] Hill MV, McMahon ML, Stucke RS, Barth RJ Jr Wide variation and excessive dosage of opioid prescriptions for common general surgical procedures. Ann Surg. 2017;265(4):709-714. doi:10.1097/SLA.0000000000001993 27631771

[12] Greco MT, Roberto A, Corli O, Quality of cancer pain management: an update of a systematic review of undertreatment of patients with cancer. J Clin Oncol. 2014;32(36):4149-4154. doi:10.1200/JCO.2014.56.0383 25403222

[13] Tanabe P, Artz N, Mark Courtney D, Adult emergency department patients with sickle cell pain crisis: a learning collaborative model to improve analgesic management. Acad Emerg Med. 2010;17(4):399-407. doi:10.1111/j.1553-2712.2010.00693.x 20370779

[14] Sinha CB, Bakshi N, Ross D, Krishnamurti L Management of chronic pain in adults living with sickle cell disease in the era of the opioid epidemic: a qualitative study. JAMA Netw Open. 2019;2(5):e194410. doi:10.1001/jamanetworkopen.2019.4410 31125105 PMC6632133

[15] Health Affairs. Optum Labs: building a novel node in the learning health care system. Accessed April 7, 2020. https://www.healthaffairs.org/doi/full/10.1377/hlthaff.2014.0038 10.1377/hlthaff.2014.0038 25006145

[16] Centers for Disease Control and Prevention. National Center for Injury Prevention and Control Data files: data files of select prescription medications, including opioids with estimated oral morphine milligram equivalent (MME) conversion factors, 2018 version. CDC compilation of benzodiazepines, muscle relaxants, stimulants, zolpidem, and opioid analgesics with oral morphine milligram equivalent conversion factors, 2018 version. Accessed April 7, 2020. https://www.cdc.gov/drugoverdose/resources/data.html

[17] Washington State Agency Medical Director’s Group Interagency guideline on prescribing opioids for pain. Published June 2015. Accessed March 31, 2020. http://www.agencymeddirectors.wa.gov/Files/2015AMDGOpioidGuideline.pdf

[18] Goldenberg DL, Clauw DJ, Palmer RE, Clair AG Opioid use in fibromyalgia: a cautionary tale. Mayo Clin Proc. 2016;91(5):640-648. doi:10.1016/j.mayocp.2016.02.002 26975749

[19] Qaseem A, Wilt TJ, McLean RM, Forciea MA; Clinical Guidelines Committee of the American College of Physicians Noninvasive treatments for acute, subacute, and chronic low back pain: a clinical practice guideline from the American College of Physicians. Ann Intern Med. 2017;166(7):514-530. doi:10.7326/M16-2367 28192789

[20] American College of Occupational and Environmental Medicine Opioids. Published by the Reed Group; 2017.

[21] Delgado MK, Huang Y, Meisel Z, National variation in opioid prescribing and risk of prolonged use for opioid-naive patients treated in the emergency department for ankle sprains. Ann Emerg Med. 2018;72(4):389-400. doi:10.1016/j.annemergmed.2018.06.003 30054152 PMC6319263

[22] American Dental Association Policy on Opioid Prescribing, 2018 Accessed March 31, 2020. https://www.ada.org/en/advocacy/current-policies/substance-use-disorders

[23] Washington State Agency Medical Director’s Group Dental guideline on prescribing opioids for acute pain management. Published September 2017 Accessed March 31,2020. http://www.agencymeddirectors.wa.gov/Files/20171026FINALDentalOpioidRecommendations_Web.pdf

[24] Von Korff M, Saunders K, Thomas Ray G, De facto long-term opioid therapy for noncancer pain [published correction appears in *Clin J Pain*. 2014;30(9):830]. Clin J Pain. 2008;24(6):521-527. doi:10.1097/AJP.0b013e318169d03b 18574361 PMC3286630

[25] Martin BC, Fan MY, Edlund MJ, Devries A, Braden JB, Sullivan MD Long-term chronic opioid therapy discontinuation rates from the TROUP study. J Gen Intern Med. 2011;26(12):1450-1457. doi:10.1007/s11606-011-1771-0 21751058 PMC3235603

[26] Dowell D, Haegerich TM Changing the conversation about opioid tapering. Ann Intern Med. 2017;167(3):208-209. doi:10.7326/M17-1402 28715842 PMC6608715

[27] Michigan Opioid Prescribing Engagement Network Prescribing recommendations. Accessed April 17, 2020. https://michigan-open.org/prescribing-recommendations/

[28] Thiels CA, Anderson SS, Ubl DS, Wide variation and overprescription of opioids after elective surgery. Ann Surg. 2017;266(4):564-573. doi:10.1097/SLA.0000000000002365 28697049

[29] Howard R, Fry B, Gunaseelan V, Association of opioid prescribing with opioid consumption after surgery in Michigan. JAMA Surg. 2019;154(1):e184234. doi:10.1001/jamasurg.2018.4234 30422239 PMC6439853

[30] Schmidt P, Berger MB, Day L, Swenson CW Home opioid use following cesarean delivery: how many opioid tablets should obstetricians prescribe? J Obstet Gynaecol Res. 2018;44(4):723-729. doi:10.1111/jog.13579 29359386 PMC5893418

[31] Horton JD, Munawar S, Corrigan C, White D, Cina RA Inconsistent and excessive opioid prescribing after common pediatric surgical operations. J Pediatr Surg. 2019;54(7):1427-1431. doi:10.1016/j.jpedsurg.2018.07.002 30057208

[32] Hill MV, Stucke RS, Billmeier SE, Kelly JL, Barth RJ Jr Guideline for discharge opioid prescriptions after inpatient general surgical procedures. J Am Coll Surg. 2018;226(6):996-1003. doi:10.1016/j.jamcollsurg.2017.10.012 29198638

[33] Cleeland CS, Gonin R, Hatfield AK, Pain and its treatment in outpatients with metastatic cancer. N Engl J Med. 1994;330(9):592-596. doi:10.1056/NEJM199403033300902 7508092

[34] National Comprehensive Cancer Network Adult cancer pain, version 3.2019, NCCN: clinical practice guidelines in oncology. Accessed March 31, 2020. https://jnccn.org/view/journals/jnccn/17/8/article-p977.xml 10.6004/jnccn.2019.0038 31390582

[35] Ballas SK, Kanter J, Agodoa I, Opioid utilization patterns in United States individuals with sickle cell disease. Am J Hematol. 2018;93(10):E345-E347. doi:10.1002/ajh.25233 30051929

[36] National Heart, Lung, and Blood Institute, National Institutes of Health Evidence-based management of sickle cell disease: expert panel report, 2014 Published September 2014. Accessed March 31, 2020. https://www.nhlbi.nih.gov/health-topics/evidence-based-management-sickle-cell-disease

[37] Dowell D, Haegerich T, Chou R No shortcuts to safer opioid prescribing. N Engl J Med. 2019;380(24):2285-2287. doi:10.1056/NEJMp1904190 31018066 PMC6573011

[38] Heyward J, Jones CM, Compton WM, Coverage of nonpharmacologic treatments for low back pain among US public and private insurers. JAMA Netw Open. 2018;1(6):e183044. doi:10.1001/jamanetworkopen.2018.3044 30646222 PMC6324451

