# Supplementary material for: Indication-Specific Opioid Prescribing for US Patients With Medicaid or Private Insurance, 2017
Source: JAMA Netw Open. 2020 May 11;3(5):e204514. doi: 10.1001/jamanetworkopen.2020.4514 (PMC7215258; doi:10.1001/jamanetworkopen.2020.4514)
Supplement: Supplement. — eAppendix 1. Supplementary Methods eAppendix 2. Indications Associated With Pain eTable 1. Summary Statistics of Privately Insured Population by Pain Indication, 2017 eTable 2. Summary Statistics of Medicaid Population by Pain Indication, Q4 2016 to Q3 2017 eTable 3. Opioid Prescribing Rates and Amounts for Postsurgical Pain Management Among Patients on Long-term Opioid Therapy in the United States, by Indication and Insurance Type, 2017 eTable 4. Opioid Prescribing Rates and Amounts for Nonsurgical Acute Pain Among Privately Insured Patients Not on Long-term Opioid Therapy in the United States, by Indication and Age Group, 2017 eTable 5. Opioid Prescribing Rates and Amounts for Chronic Noncancer Pain Among Privately Insured Patients in the United States, by Indication, History of Opioid Usage, and Age Group, 2017 eTable 6. Opioid Prescribing Rates and Amounts for Postsurgical Pain Management Among Privately Insured Patients Not on Long-term Opioid Therapy in the United States, by Indication and Age Group, 2017 eTable 7. Opioid Prescribing Rates and Amounts for Nonsurgical Acute Pain Among Medicaid Enrollees Not on Long-term Opioid Therapy in the United States, by Age Group, Q4 2016–Q3 2017 eTable 8. Opioid Prescribing Rates and Amounts for Chronic Noncancer Pain Among Medicaid Enrollees in the United States, by Indication, Timing of Chronic Pain Diagnosis, History of Opioid Usage, and Age Group, Q4 2016–Q3 2017 eTable 9. Opioid Prescribing Rates and Amounts for Postsurgical Pain Management Among Medicaid Enrollees Not on Long-term Opioid Therapy in the United States, by Indication and Age Group, Q4 2016–Q3 2017 eReferences. [file jamanetwopen-3-e204514-s001.pdf]

Supplementary Online Content

Mikosz CA, Zhang K, Haegerich T, et al. Indication-specific opioid prescribing for US patients with Medicaid or private insurance, 2017. *JAMA Netw Open*. 2020;3(5):e204514. doi:10.1001/jamanetworkopen.2020.4514

eAppendix 1. Supplementary Methods
eAppendix 2. Indications Associated With Pain
eTable 1. Summary Statistics of Privately Insured Population by Pain Indication, 2017
eTable 2. Summary Statistics of Medicaid Population by Pain Indication, Q4 2016 to Q3 2017
eTable 3. Opioid Prescribing Rates and Amounts for Postsurgical Pain Management Among Patients on Long-term Opioid Therapy in the United States, by Indication and Insurance Type, 2017
eTable 4. Opioid Prescribing Rates and Amounts for Nonsurgical Acute Pain Among Privately Insured Patients Not on Long-term Opioid Therapy in the United States, by Indication and Age Group, 2017
eTable 5. Opioid Prescribing Rates and Amounts for Chronic Noncancer Pain Among Privately Insured Patients in the United States, by Indication, History of Opioid Usage, and Age Group, 2017
eTable 6. Opioid Prescribing Rates and Amounts for Postsurgical Pain Management Among Privately Insured Patients Not on Long-term Opioid Therapy in the United States, by Indication and Age Group, 2017
eTable 7. Opioid Prescribing Rates and Amounts for Nonsurgical Acute Pain Among Medicaid Enrollees Not on Long-term Opioid Therapy in the United States, by Age Group, Q4 2016–Q3 2017
eTable 8. Opioid Prescribing Rates and Amounts for Chronic Noncancer Pain Among Medicaid Enrollees in the United States, by Indication, Timing of Chronic Pain Diagnosis, History of Opioid Usage, and Age Group, Q4 2016–Q3 2017
eTable 9. Opioid Prescribing Rates and Amounts for Postsurgical Pain Management Among Medicaid Enrollees Not on Long-term Opioid Therapy in the United States, by Indication and Age Group, Q4 2016–Q3 2017
eReferences.

This supplementary material has been provided by the authors to give readers additional information about their work.

© 2020 Mikosz CA et al. *JAMA Network Open*.

eAPPENDIX 1. Supplementary Methods

Structure and engagement of the National Center of Injury Prevention and Control Board of Scientific Counselors Opioid Prescribing Estimates Workgroup

CDC presented the study research protocol to the National Center for Injury Prevention and Control Board of Scientific Counselors (NCIPC BSC), which provided recommendations to CDC with input from an Opioid Prescribing Estimates (OPE) Workgroup. The OPE Workgroup was comprised of experts from a variety of specialties, including dentistry, internal medicine, family medicine, pediatrics, emergency medicine, general surgery and surgical subspecialties (e.g., orthopedic surgery, plastic surgery, transplant surgery, surgical oncology, trauma surgery), pain medicine/anesthesiology, obstetrics/gynecology/urology, oncology, palliative medicine, hematology, physical medicine/rehabilitation, behavioral health, occupational medicine, pharmacy, geriatrics, and neurology. Experts had extensive scientific expertise, clinical training, and/or experience in opioid prescribing and pain management. Both physicians and non-physician practitioners were included, as well as two patient representatives and a bioethicist. Experts declared interests consistent with advisory committee conflict of interest protocols.

The OPE Workgroup met via closed teleconference four times during September to October 2018 to discuss the scope of this analysis, application of clinical research, policy implications, and prioritization of clinical indications for study inclusion. OPE Workgroup meeting discussions were summarized in a Workgroup report that was sent to the NCIPC BSC in early December 2018. During their December 12, 2018 meeting, the NCIPC BSC provided their recommendations to CDC regarding the direction of this project based on review of the OPE Workgroup report. The OPE Workgroup report and the minutes of the December 12, 2018 NCIPC BSC meeting can be found at https://www.cdc.gov/injury/bsc/meetings.html.

Core members of the OPE Workgroup included: Phillip Coffin, MD, MIA (Workgroup Chair); Roger Chou, MD; Chinazo Cunningham, MD, MS; Christina Porucznik, PhD, MSPH; Lonnie Zeltzer, MD; Jennifer Waljee, MD, MPH; Jeanmarie Perrone, MD; Mark Wallace, MD; Carolyn Swenson, MD; Judith Paice, PhD, RN; Amanda Brandow, DO; M. Cary Reid, MD; Elizabeth Eckstrom, MD, MPH; Travis Rieder, PhD (bioethicist); Penney Cowan (patient representative); Joan Maxwell (patient representative); and Steven Stanos, DO. A quorum of core members was required to be present at all Workgroup meetings.

Ad hoc members of the OPE Workgroup included: Yngvild Olsen, MD, MPH; G. Caleb Alexander, MD, MS; Erin Krebs, MD, MPH; Mallika Mundkur, MD, MPH; Dwight Humpherys, DO; Thomas Weiser, MD; William Zempsky, MD, MPH; Raeford Brown, MD; Renee Manworren, PhD, MS; Adil Haider, MD, MPH; Elizabeth Habermann, PhD; David Ring, MD, PhD; Richard Barth, MD; Hassan Mir, MD, MBA; Michael Englesbe, MD; Lewis Nelson, MD; Mark Rosenberg, DO, MBA; M. Kit Delgado, MD; Chad Brummett, MD; Greg Terman, MD, PhD; Brian Bateman, MD, MSc; Mikio Nihira, MD, MPH; Carolyn Lefkowits, MD, MPH; Joshua Field, MD, MS; Raj Mitra, MD; Judith Turner, PhD; Wilson Compton, MD, MPE; Paul Moore, DMD, PhD, MS, MPH; Harold Tu, MD, DMD; Elliot Hersh, DMD, MS, PhD; Raymond Dionne, DDS, MS, PhD; David Dodick, MD; Benjamin Friedman, MD; Suzanne Nesbit, PharmD; and Kurt Hegmann, MD, MPH. Ad hoc members could attend any of the scheduled meetings; however, meetings were arranged to maximize attendance of ad hoc members with expertise/medical specialty relevant to the meeting topic being discussed.

Members of the NCIPC Board of Scientific Counselors at the time of the December 12, 2018 meeting included: Donna H. Barnes, PhD; Phillip Coffin, MD, MIA; R. Dawn Comstock, PhD; Kermit Crawford, PhD; Chinazo Cunningham, MD, MS; Elizabeth Eckstrom, MD, MPH; Frank A. Franklin, PhD, JD, MPH; Victoria Frye, PhD; James Hedlund, PhD; Todd Herrenkohl, PhD; Mark S. Kaplan, DrPH; Karen D. Liller, PhD: David C. Schwebel, PhD; Debora Daro-Tuggle; Federico Vaca, MD, MPH; Daniel J. Whitaker, PhD. Ex-Officio members: Rory Austin, PhD; Melissa Brodowski, PhD, MSW, MPH; Dawn Castillo, MPH; Mindy Chai, JD, PhD; Wilson Compton, MD, MPH; Holly Hedegaard, MD, MSPH; Calvin Johnson; Lyndon Joseph, PhD; Amy Leffler, PhD; Valerie Maholmes, PhD, CAS; Constantinos Miskis, JD; Thomas Schroeder, MS; and RADM Kelly Taylor, MPH.

Data sources

The OptumLabs Data Warehouse (OLDW) administrative claims data includes adjudicated claims for all healthcare services incurred by enrollees. All claims data are presented by OLDW as professional claims (office visits, physician services at outpatient and inpatient hospitals), facility claims (facility services at outpatient and inpatient hospitals), and prescription drug claims. The data also includes beneficiaries' enrollment details and demographic information. Professional and facility claims contain information such as International Classification of Diseases, 10th Revision, Clinical Modification (ICD-10-CM) diagnosis codes (up to four diagnoses on each professional claim and nine on each facility claim), Current Procedural Terminology (CPT) codes, place of service codes, provider specialty codes, de-identified patient and provider identification

number (ID), and service date. Prescription drug claims contain National Drug Code (NDC), days of supply, quantity dispensed, de-identified patient and prescriber ID, and dispensing date. All census regions are represented in the OLDW, although there is a higher proportion of OLDW beneficiaries in the Southern and Midwestern census regions than in the entire insured population.

Similar to OLDW claims data, the MarketScan Multi-State Medicaid Database (MMD) contains comprehensive adjudicated claims data across the care continuum, i.e. physician office visits, outpatient hospital, inpatient hospital, and prescription drugs. The data set also includes Medicaid beneficiaries' enrollment details and demographic information. Outpatient and inpatient claims contain similar information as the OLDW data, including ICD-10-CM diagnosis codes (up to four diagnoses on outpatient claims), CPT codes, de-identified patient ID, and service date. Prescription drug claims contain National Drug Codes (NDC), days of supply, quantity dispensed, de-identified patient ID, and dispensing date.

We had access to the entire population included in the OLDW dataset and the MMD. We also had access to all health insurance claims contained within these two datasets, including outpatient claims, inpatient claims, physician office visits, pharmaceutical prescription claims, and the enrollment file. The OLDW dataset and the MMD are cleaned for research use by the data vendor, i.e. OptumLabs and IBM, respectively. We accessed OLDW claims data through the secured OptumLabs data portal. The MMD was delivered to CDC and stored on a secured network drive. We used the data directly to identify our study cohorts.

For both datasets, the overall study sample included all beneficiaries enrolled with both medical and prescription drug coverage for at least ten continuous months from July 1, 2016—March 31, 2018 (OLDW) and April 1, 2016—December 31, 2017 (MMD). These differing windows reflect the availability of only 2016 and 2017 MMD data for this analysis. Ten months of continuous enrollment comprised the month of the index diagnosis, including at least six months prior and at least three months after it (to determine whether the diagnosis was acute or persisted as chronic). Thus, the continuous enrollment period varied by individual enrollee depending on the date of the index diagnosis.

Indication inclusion criteria

Indications were chosen for analysis based on a combination of their association with opioid prescribing reported in the literature; feedback from the Opioid Prescribing Estimates Workgroup via the NCIPC BSC regarding clinical importance; and frequency in the OLDW dataset (i.e., rare conditions and procedures were excluded). Claims that contained the ICD-10-CM code for "Z51.5 encounter for palliative care" were excluded from analysis.

Definitions of indications associated with pain

Indications associated with nonsurgical acute pain

We included abdominal pain, acute low back pain, acute migraine, dental pain, rib fractures, herpes zoster, renal colic, and musculoskeletal sprains/strains (ICD-10-CM codes are listed in eAppendix 2). For each acute pain indication, the diagnosis code itself is insufficient to determine whether the indication is of an acute or chronic nature. Thus, we first used ICD-10-CM diagnosis codes in professional claims from January 1, 2017—December 31, 2017 (OLDW) or October 1, 2016—September 30, 2017 (MMD) to identify all visits with a particular nonsurgical acute pain indication. Second, we excluded all identified visits that represented inpatient stays. Third, we defined the first diagnosis of a specific nonsurgical acute pain indication in 2017 (or starting with October 1, 2016, for MMD data) as the "index diagnosis" for a patient. Fourth, we used six months of claims prior to the index diagnosis to determine whether it was a new or pre-existing diagnosis. Fifth, we used claims three months after the "index diagnosis" to determine whether the index diagnosis persisted beyond this timeframe. Sixth, we only included visits that met the following criteria: 1) a new diagnosis, i.e. no same diagnosis in the six months prior, as determined during the fourth step; 2) a diagnosis that persisted three months or less, as determined during the fifth step; 3) no more than three visits for the same diagnosis within the three months following the "index diagnosis" during the study period. There was one exception in approach: in order to broadly capture abdominal pain complaints, we used the nonspecific abdominal pain symptom ICD-10-CM codes (R100, R101, R103, or R109) on a claim record to identify these visits.

Indications associated with chronic pain

We included non-radicular back pain, radicular back pain, neck pain, fibromyalgia, inflammatory joint disorders, irritable bowel syndrome, non-migraine headaches, osteoarthritis and joint cartilage conditions, and periarticular/soft tissue disorders (ICD-10-CM codes are in eAppendix 2). Similar to nonsurgical acute pain indications, the diagnosis code itself is insufficient to determine whether the indication is of a chronic nature. To identify chronic pain indications for our analysis, we first applied a similar process to that described earlier for nonsurgical acute pain. Thus, we first used ICD-10-CM diagnosis codes in professional claims from January 1, 2017—December 31, 2017 (OLDW) or October 1, 2016—September 30, 2017 (MMD) to identify all visits with a particular chronic pain indication. Second, we excluded all identified visits that represented inpatient stays. Third, we defined the first diagnosis of a specific chronic pain indication in 2017 (or starting with

© 2020 Mikosz CA et al. *JAMA Network Open*.

October 1, 2016, for MMD data) as the “index diagnosis” for a patient. Fourth, we used six months of claims prior to the index diagnosis to determine whether it was a new or pre-existing diagnosis. Fifth, we used claims three months after the “index diagnosis” to determine whether the index diagnosis persisted beyond this timeframe. However, different from the approach for nonsurgical acute pain, we then only included in our analysis those visits for which the chronic pain indication persisted beyond three months after the index diagnosis, as determined in the fifth step[1,2].

Indications associated with postsurgical pain

We included 24 surgical procedures in our study (the list of procedures and their CPT codes are in eAppendix 2). Surgical procedures were identified by using the CPT code on professional claims, i.e. from providers who performed procedures. We included professional claims from January 1, 2017—December 31, 2017 (OLDW) or October 1, 2016—September 30, 2017 (MMD) from both outpatient and inpatient settings in order to capture procedures occurring in both settings.

SCD pain

SCD pain was analyzed separately from the other pain categories due to the inability to reliably differentiate between acute pain related to a sickle cell vaso-occlusive crisis and chronic SCD-related pain using claims data and because of the unique clinical characteristics and approach to pain management for this patient population. To analyze opioid prescriptions for pain related to SCD, we first applied the same steps as listed above for chronic pain. Then, as per prior studies[3,4] we only included those patients who had at least three distinct visits with an ICD-10-CM code for SCD during the study period (see eAppendix 2 for a list of ICD-10-CM codes).

Cancer pain

We analyzed opioid prescriptions written for cancer pain separately from the other categories above, due to the unique clinical characteristics and approach to pain management for this patient population. Cancer patients were identified as having at least two visits in January 1, 2017—December 31, 2017 (OLDW) or October 1, 2016—September 30, 2017 (MMD) with both a cancer diagnosis and a provider specialty listed as “oncologist” or “oncology.” ICD-10-CM codes for this indication are listed in eAppendix 2.

Opioid prescription linkage methodology

Indications associated with postsurgical pain

For surgical procedures, we linked an opioid prescription to a procedure if the prescription met all of the following criteria: 1) the prescription and procedure had the same patient ID; 2) the prescription was dispensed within 5 days after the procedure including the day of procedure; and 3) there were no additional procedures or visits between the procedure in question and the date the prescription was issued.

Given the possibility of patients obtaining opioid prescriptions from surgeons prior to a scheduled procedure, we also applied additional criteria to link prescriptions to procedures: 1) the prescription and procedure had the same patient ID; 2) the prescription was dispensed within 30 days prior to the procedure; 3) the prescriber specialty was “surgeon;” and 4) the prescription was not linked to any other procedure or visit prior to the date of this prescription.

Indications associated with nonsurgical acute pain

For nonsurgical acute pain indications, we linked an opioid prescription to a visit if the prescription met all of the following criteria: 1) the prescription was not linked to any surgical procedure; 2) the prescription and visit had the same patient ID; 3) prescription was dispensed within 7 days after the visit, including the day of visit; and 4) there were no additional visits with the diagnosis in question between this visit and the date the prescription was issued.

To further ensure the accurate linkage of opioid prescriptions to nonsurgical acute pain indications, we searched for a patient’s opioid prescriptions, if any, in the three months prior to his or her visit where the index diagnosis was made. A patient receiving long-term opioid therapy (LTOT) was defined as: 1) having at least three opioid prescriptions in the three months prior to the index visit; 2) more than 60 total days of opioid supply; and 3) the gap between the end of one prescription and the next prescription was fewer than 10 days. If a patient was identified as receiving LTOT prior to the index acute pain diagnosis, we excluded the patient from the nonsurgical acute pain category to increase confidence that a given opioid prescription was for the nonsurgical acute indication, since a patient with a new nonsurgical acute condition may present to the same clinician that prescribes LTOT for a chronic indication.

We conducted our analysis for nonsurgical acute pain at the visit level because of the nature of acute pain conditions, for which each presentation for care may represent distinct events. The majority of patients in this category had a single visit (i.e. the index diagnosis visit) for an acute pain condition between the date of index diagnosis and the three months following the

© 2020 Mikosz CA et al. *JAMA Network Open*.

index visit. However, in cases when a patient had more than one visit of the same nonsurgical acute pain condition, each visit was counted separately. When a prescription was linked to more than one nonsurgical acute pain indication, we first applied the provider ID and provider specialty, where it was available, to further assign the prescription. When it was not available, we assigned the prescription to the indication whose visit date was closer to the prescription.

Indications associated with chronic pain or SCD pain

For chronic pain indications and SCD pain, we linked an opioid prescription to a visit if the prescription met all of the following criteria: 1) the prescription was not linked to any surgical procedure or nonsurgical acute pain; 2) the prescription and visit had the same patient ID; 3) the prescription was dispensed within 14 days after the visit including the day of visit; and 4) there were no additional visits with the diagnosis in question between this visit and the date the prescription was issued. The wider window of 14 days was used because the filling of opioid prescriptions for chronic pain indications might not occur as rapidly as for acute pain, possibly because a patient might already have opioids on hand. In cases where a patient had two concurrent chronic or SCD pain indications that overlapped within the same time frame, the opioid prescription was assigned based on the likelihood tier to which the indication was assigned (see below). These likelihood tiers were constructed based on multiple published clinical guidelines[5-10] and, based on that published guidance, the likelihood that an opioid would be prescribed for that condition if guideline recommendations were followed. If two indications fell in the same tier, the prescription was assigned to whichever indication was always coded as the primary diagnosis. If primary indication could not be identified, the prescription was assigned to each indication.

**INDICATION ASSOCIATED WITH PAIN**	**LIKELIHOOD OF OPIOID PRESCRIPTION**
Chronic nonradicular back pain	A
Chronic radicular back pain	A
Chronic neck pain	A
Fibromyalgia	B
Inflammatory joint disorders	B
Irritable bowel syndrome	B
Non-migraine headaches	B
Osteoarthritis and joint cartilage conditions	A
Periarticular/soft tissue disorders	B
Sickle cell disease	A

A = Opioids may be prescribed. Opioids may be considered for pain management if benefits are felt to outweigh risks, based on existing clinical guidelines.

B = Opioids not likely to be prescribed. Based on existing clinical guidelines, opioids are not typically used for pain management as the risks are usually felt to outweigh the benefits.

Criteria to identify patients receiving LTOT are described above under "Indications associated with nonsurgical acute pain." A patient not receiving LTOT in our analysis was any patient that did not meet the criteria for being on LTOT.

Cancer pain

For cancer-related pain, opioid prescriptions were linked following a similar methodology to that described above for chronic pain indications and SCD-related pain. However, the prescription linkage for this category occurred as the primary step in the linkage algorithm, meaning that opioid prescriptions linked to management of cancer pain were thus excluded from the linkage process for other pain indications.

© 2020 Mikosz CA et al. *JAMA Network Open*.

Opioid prescription linkage algorithm

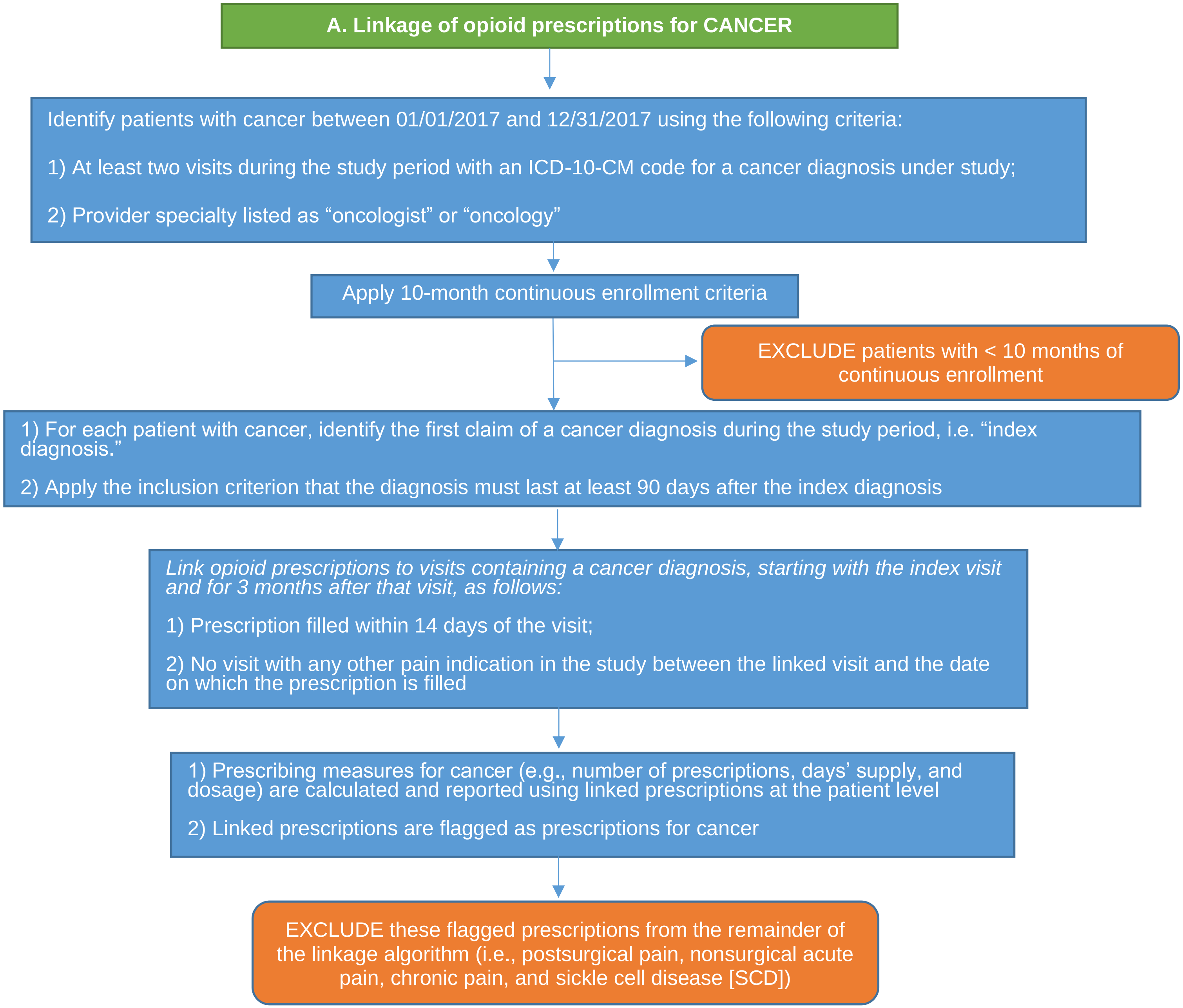

© 2020 Mikosz CA et al. *JAMA Network Open*.

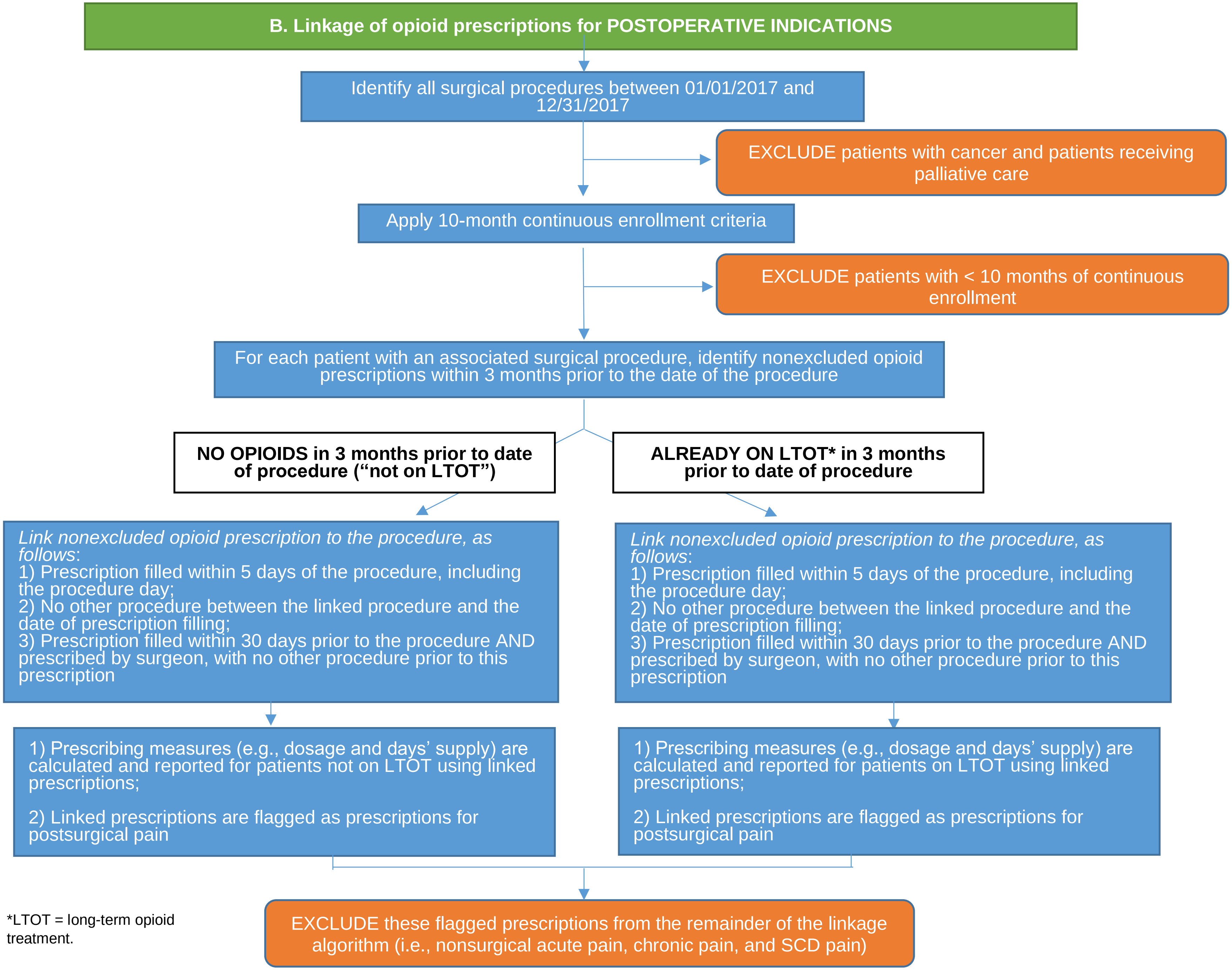

*LTOT = long-term opioid treatment.

© 2020 Mikosz CA et al. *JAMA Network Open*.

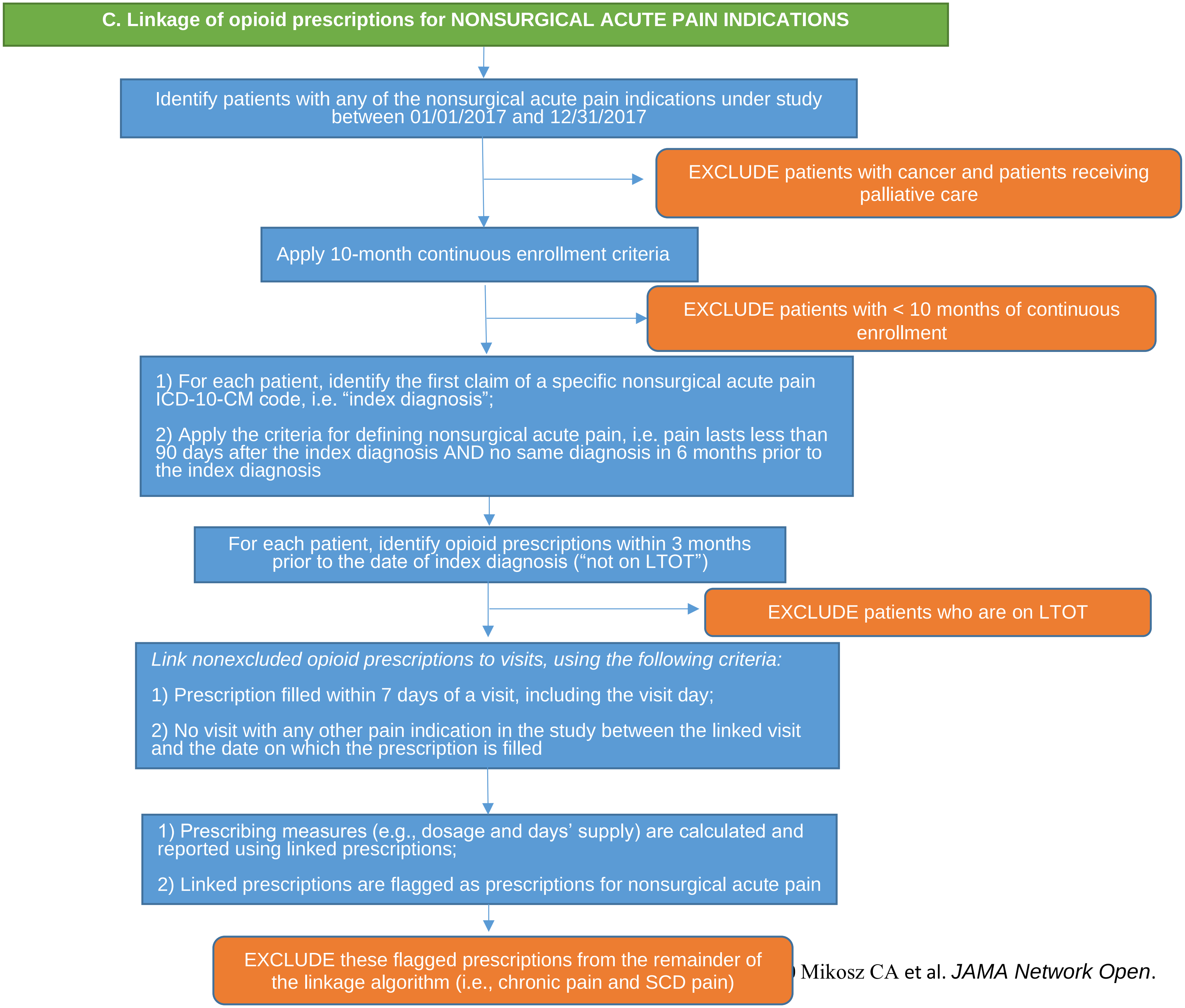
C. Linkage of opioid prescriptions for NONSURGICAL ACUTE PAIN INDICATIONS
Identify patients with any of the nonsurgical acute pain indications under study between 01/01/2017 and 12/31/2017
EXCLUDE patients with cancer and patients receiving palliative care
Apply 10-month continuous enrollment criteria
EXCLUDE patients with < 10 months of continuous enrollment
1) For each patient, identify the first claim of a specific nonsurgical acute pain ICD-10-CM code, i.e. “index diagnosis”;
2) Apply the criteria for defining nonsurgical acute pain, i.e. pain lasts less than 90 days after the index diagnosis AND no same diagnosis in 6 months prior to the index diagnosis
For each patient, identify opioid prescriptions within 3 months prior to the date of index diagnosis (“not on LTOT”)
EXCLUDE patients who are on LTOT
Link nonexcluded opioid prescriptions to visits, using the following criteria:
1) Prescription filled within 7 days of a visit, including the visit day;
2) No visit with any other pain indication in the study between the linked visit and the date on which the prescription is filled
1) Prescribing measures (e.g., dosage and days’ supply) are calculated and reported using linked prescriptions;
2) Linked prescriptions are flagged as prescriptions for nonsurgical acute pain
EXCLUDE these flagged prescriptions from the remainder of the linkage algorithm (i.e., chronic pain and SCD pain)

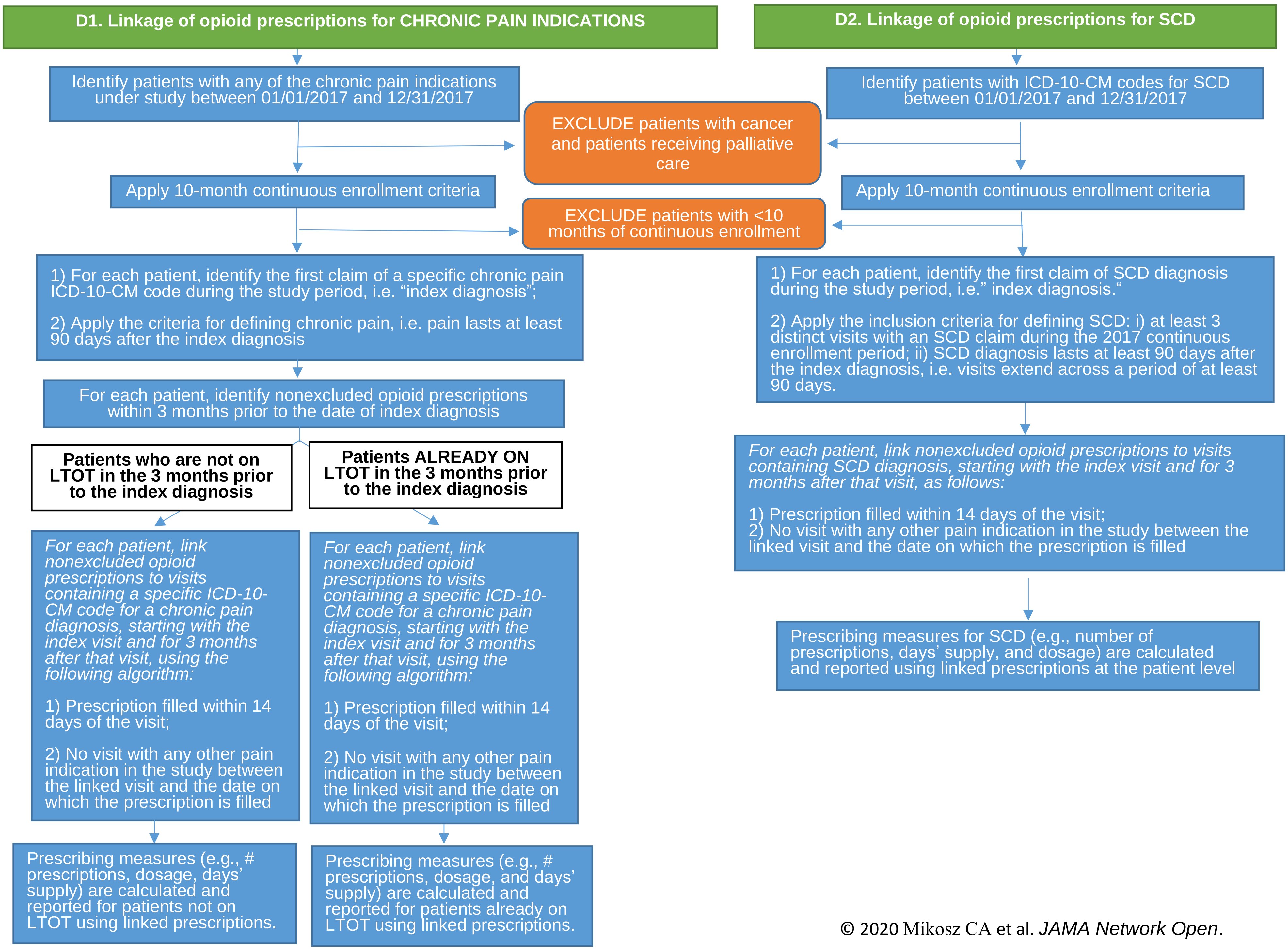

© 2020 Mikosz CA et al. *JAMA Network Open*.

eAPPENDIX 2. Indications Associated With Pain

ICD-10 CM codes for indications associated with nonsurgical acute pain

**Abdominal pain**<br><br>R100, R101, R103, R109
**Acute low back pain**<br><br>Q762, M4300, M4301, M4302, M4303, M4304, M4305, M4306, M4307, M4308, M4309, M4310, M4311, M4312, M4313, M4314, M4315, M4316, M4317, M4318, M4319, M545, M5430, M5431, M5432, M5440, M5441, M5442, M5114, M5115, M5116, M5117, M5414, M5415, M5416, M5417, M5489, M549, M4327, M4328, M532X7, M532X8, M533, M5386, M5387, M5388, M5403, M5404, M5405, M5406, M5407, M5408, M5409, M62830, M4320, M4321, M4322, M4323, M4324, M4325, M4326, M438X9, M5380, M5384, M5385, M539, M4820, M4821, M4822, M4823, M4824, M4825, M4826, M4827, M4726, M4727, M4728, M47816, M47817, M47818, M47896, M47897, M47898
**Acute migraine**<br><br>G43001, G43009, G43011, G43019, G43101, G43109, G43111, G43119, G43C0, G43B0, G43D0, G43A0, G43A1, G43C1, G43B1, G43D1, G43401, G43409, G43411, G43419, G43501, G43509, G43511, G43519, G43609, G43619, G43601, G43611, G43701, G43711, G43709, G43719, G43801, G43811, G43809, G43819, G43901, G43911, G43909, G43919, G43821,<br>G43829, G43831, G43839
**Dental pain**<br><br>K006, K007, K010, K011, K023, K0252, K0253, K0262, K0263, K027, K029, K030, K031, K032, K0381, K040, K0401, K0402, K044, K046, K047, K08109, K089
**Herpes zoster**<br><br>B02
**Rib fractures**<br><br>S223, S224
**Musculoskeletal sprains and strains**<br><br>S4352XA, S4351XA, S4350XA, S43411A, S43412A, S43419A, S43422A, S43429A, S43421A, S4380XA, S4381XA, S4382XA, S43432A, S43431A, S43439A, S4362XA, S46811A, S46819A, S46812A, S46212A, S4361XA, S43492A, S46011A, S46019A, S43499A, S4360XA, S43491A, S46111A, S46112A, S46319A, S46211A, S46312A, S46219A, S46012A, S46311A, S46119A, S43401A, S43402A, S4392XA, S43409A, S4390XA, S46919A, S46912A, S4391XA, S46911A, S53432A, S5322XA, S5321XA, S53431A, S5320XA, S53439A, S53441A, S53442A, S5330XA, S5331XA, S53449A, S5332XA, S53412A, S53419A, S53411A, S53422A, S53429A, S53421A, S53491A, S53492A, S53499A, S56114A, S56411A, S56413A, S53401A, S56115A, S56415A, S56312A, S56416A, S56417A, S56319A, S56019A, S56116A, S56912A, S56414A, S53402A, S56511A, S56011A, S56111A, S56117A, S56119A, S56212A, S56211A, S56112A, S56113A, S56911A, S56219A, S56512A, S56118A, S56819A, S56418A, S56519A, S56811A, S56012A, S56412A, S56812A, S56419A, S53409A, S56311A, S56919A, S66919A, S66911A, S66912A, S63502A, S63509A, S63501A, S63512A, S63511A, S63519A, S63329A, S63521A, S63529A, S63522A, S63321A, S63322A, S66115A, S66514A, S66512A, S66118A, S66511A, S66311A, S66519A, S66113A, S66219A, S66318A, S66019A, S66212A, S66314A, S66117A, S63392A, S66515A, S66518A, S66110A, S66516A, S63339A, S66119A, S63309A, S66412A, S66211A, S66812A, S66510A, S66811A, S66517A, S63311A, S63331A, S63301A, S66419A, S66012A, S66114A, S66312A, S63391A, S63599A, S63319A, S63591A, S66411A, S63399A, S66313A, S66011A, S66315A, S63332A, S63302A, S66319A, S66111A, S66310A, S66819A, S66112A, S63592A, S66116A, S66513A, S66317A, S66316A, S63312A, S6392XA, S6390XA, S6391XA,

© 2020 Mikosz CA et al. *JAMA Network Open*.

S63659A, S63651A, S63654A, S63641A, S63657A, S63655A, S63658A, S63650A, S63642A, S63652A, S63649A, S63653A, S63656A, S63636A, S63621A, S63631A, S63629A, S63630A, S63639A, S63633A, S63635A, S63632A, S63637A, S63634A, S63622A, S63638A, S63411A, S63490A, S63408A, S63696A, S638X1A, S63425A, S63615A, S63415A, S63431A, S63611A, S63694A, S63438A, S63409A, S63406A, S63691A, S63424A, S63498A, S63610A, S63434A, S63433A, S63609A, S63617A, S63495A, S63414A, S63690A, S63494A, S63699A, S638X9A, S63429A, S63430A, S63404A, S63496A, S63614A, S63427A, S63422A, S63403A, S63689A, S63420A, S63493A, S63619A, S63499A, S63421A, S63439A, S63410A, S63618A, S63405A, S63416A, S63697A, S63418A, S63428A, S63401A, S63436A, S63407A, S63698A, S63612A, S63435A, S63695A, S63417A, S63682A, S63402A, S638X2A, S63616A, S63437A, S63613A, S63681A, S63432A, S63426A, S63413A, S63497A, S63491A, S63419A, S63601A, S63412A, S63492A, S63400A, S63693A, S63423A, S63692A, S63602A, S73111A, S73119A, S73112A, S73122A, S73129A, S73121A, S76212A, S76811A, S76119A, S76812A, S73191A, S76219A, S76111A, S76019A, S73192A, S76011A, S76211A, S76112A, S76012A, S76311A, S76819A, S73199A, S76312A, S76319A, S76919A, S73101A, S73109A, S76911A, S73102A, S76912A, S83429A, S83422A, S83421A, S83419A, S83412A, S83411A, S83512A, S83521A, S83529A, S83522A, S83502A, S83519A, S83501A, S83511A, S83509A, S8362XA, S8361XA, S8360XA, S83401A, S86811A, S86212A, S86112A, S86111A, S86819A, S86211A, S86311A, S86319A, S86312A, S86119A, S83402A, S86219A, S83409A, S838X9A, S86812A, S838X2A, S838X1A, S86912A, S8390XA, S8392XA, S8391XA, S86911A, S86919A, S93402A, S96919A, S93401A, S93409A, S93421A, S93422A, S93429A, S93411A, S93419A, S93412A, S93432A, S93439A, S93431A, S96212A, S96911A, S96011A, S96012A, S93491A, S96119A, S96811A, S96112A, S96219A, S86019A, S96019A, S93492A, S86012A, S96819A, S86011A, S96111A, S96812A, S96912A, S93499A, S96211A, S93601A, S93602A, S93609A, S93629A, S93622A, S93621A, S93526A, S93529A, S93524A, S93525A, S93522A, S93521A, S93523A, S93519A, S93514A, S93515A, S93513A, S93512A, S93516A, S93511A, S93509A, S93619A, S93611A, S93504A, S93505A, S93612A, S93501A, S93699A, S93506A, S93503A, S93692A, S93691A, S93502A, S338XXA, S336XXA, S161XXA, S134XXA, S138XXA, S233XXA, S238XXA, S335XXA, S139XXA, S339XXA, S239XXA, S038XXA, S0342XA, S0343XA, S0340XA, S0341XA, S034XXA, S135XXA, S2341XA, S23429A, S23420A, S23421A, S23428A, S334XXA, S39012A, S29019A, S039XXA, S39013A, S39011A, S29012A, S29011A
**Renal colic**<br><br>N200, N202, N23

© 2020 Mikosz CA et al. *JAMA Network Open*.

ICD-10 CM codes for indications associated with chronic pain

**Chronic nonradicular back pain**<br><br>M2578, M4000, M4003, M4004, M4005, M40202, M40203, M40204, M40205, M40209, M40292, M40293, M40294, M40295, M40299, M4030, M4035, M4036, M4037, M4100, M4102, M4103, M4104, M4105, M4106, M4107, M4108, M41112, M41113, M41114, M41115, M41116, M41117, M41119, M41122, M41123, M41124, M41125, M41126, M41127, M41129, M4120, M4122, M4123, M4124, M4125, M4126, M4127, M4130, M4134, M4135, M4180, M4182, M4183, M4184, M4185, M4186, M4187, M419, M4300, M4301, M4302, M4303, M4304, M4305, M4306, M4307, M4308, M4309, M4310, M4311, M4312, M4313, M4314, M4315, M4316, M4317, M4318, M4319, M4320, M4321, M4322, M4323, M4324, M4325, M4326, M4327, M4328, M438X9, M4640, M4644, M4645,M4646, M4647, M4648, M4649, M4710, M4714, M4715, M4716, M4720, M47814, M47815, M47816, M47817, M47818, M47819, M47894, M47895, M47896, M47897, M47898, M47899, M479, M4800, M4804, M4805, M4806, M48061, M48062, M4807, M4808, M4810, M4811, M4812, M4813, M4814, M4815, M4816, M4817, M4818, M4819, M4820, M4821, M4822, M4823, M4824, M4825, M4826, M4827, M4830, M4831, M4832, M4833, M4834, M4835, M4836, M4837, M4838, M489, M5104, M5105, M5106, M5124, M5125, M5126, M5127, M5134, M5135, M5136, M5137, M5146, M5147, M5184, M5185, M5186, M5187, M519, M532X7, M532X8, M533, M5380, M5384, M5385, M5386, M5387, M5388, M539, M5403, M5404, M5405, M5406, M5407, M5408, M5409, M5430, M5431, M5432, M545, M546, M5489, M549, M62830, M961, M962, M963, M965, M9922, M9923, M9924, M9925, M9926, M9927, M9928, M9929, M9932, M9933, M9934, M9935, M9936, M9937, M9938, M9939, M9942, M9943, M9944, M9945, M9946, M9947, M9948, M9949, M9952, M9953, M9954, M9955, M9956, M9957, M9958, M9959, M9962, M9963, M9964, M9965, M9966, M9967, M9968, M9969, M9972, M9973, M9974, M9975, M9976, M9977, M9978, M9979, M9983, M9984, M9902, M9904, Q762
**Chronic radicular back pain**<br><br>M4724, M4725, M4726, M4727, M4728, M5114, M5115, M5116, M5117, M5414, M5415, M5416, M5417, M5418, M5440, M5441, M5442
**Chronic neck pain**<br><br>M47811, M47891, M4723, M4722, M4721, M47813, M47893, M47892, M47812, M47012, M4711, M47014, M47011, M4712, M47022, M47029, M47019, M47016, M47013, M47021, M4713, M47015, M50220, M5022, M5023, M5020, M50221, M50222, M50223, M5021, M5002, M5000, M5001, M50022, M50023, M50021, M50020, M5003, M5082, M5091, M5012, M50922, M50821, M5080, M5011, M50123, M50822, M50820, M50921, M5010, M4643, M5093, M50120, M50823, M5083, M50121, M5090, M4641, M50920, M50122, M4642, M5092, M50923, M5013, M5081, M9930, M9971, M9921, M9970, M4803, M9931, M9940, M9960, M9950, M4802, M9951, M4801, M9961, M9941, M9920, M542, M530, M531, M5413, M5411, M5412, M436, M5400, M5402, M5401, M5382, M5481, M5381, M5383
**Fibromyalgia**<br><br>M797
**Inflammatory joint disorders**<br><br>M05, M06, M08, M120, M255, M353, M45, M460, M461, M465, M468, M469, M488X, M498, M790
**Irritable bowel syndrome**<br><br>K58
**Non-migraine headaches**<br><br>G44009, G44001, G44019, G44011, G44021, G44029, G44031, G44039, G44041, G44049, G44059, G44051, G44091, G44099, G44201, G44211, G44219, G44229, G44221, R51, G441

© 2020 Mikosz CA et al. *JAMA Network Open*.

**Osteoarthritis and joint cartilage conditions**<br><br>M129, M131, M138, M15, M16, M17, M18, M19, M221, M222, M223, M224, M228, M229, M23, M241
**Periarticular/soft tissue disorders**<br><br>M7500, M7501, M7502, M2570, M2571, M2572, M2573, M2574, M2575, M2576, M2577, M66211, M66212, M66219, M66811, M66812, M66819, M701, M702, M704, M705, M706, M707, M751, M752, M753, M754, M755, M758, M759, M76, M77, M2161, M2162, M65, M66, M67, M700, M7030, M7031, M7032, M71, M7512

© 2020 Mikosz CA et al. *JAMA Network Open*.

Current Procedural Terminology (CPT) codes for surgical procedures

**Abdominal solid organ resection, laparoscopic**<br>58150, 58152, 58180, 58200, 58210, 58951, 58953, 58954, 58956, 58240
**Appendectomy, laparoscopic**<br>44950, 44955, 44960, 44970, 44979
**Arthroscopic knee surgery**<br>29882, 29883, 29888, 29889, 29891, 29892
**Arthroscopic rotator cuff repair**<br>23410, 23412, 24341, 29827
**Cesarean section**<br>59510, 59514, 59515, 59618, 59620, 59622
**Cholecystectomy, laparoscopic**<br>47562, 47563, 47564, 47570
**Cholecystectomy, open**<br>47420, 47425, 47600, 47605, 47610, 47612, 47620
**Colectomy, laparoscopic**<br>44204, 44205, 44206, 44207, 44208, 44210, 44211, 44212, 44213
**Colectomy, open**<br>44139, 44140, 44141, 44143, 44144, 44145, 44146, 44147, 44150, 44151, 44155, 44156, 44157, 44158, 44160, 45121, 45123
**Coronary artery bypass**<br>33510, 33511, 33512, 33513, 33514, 33516, 33517, 33518, 33519, 33521, 33522, 33523, 33530, 33533, 33534, 33535, 33536
**Excisional biopsy**<br>20205, 20245, 27324, 27614, 38500, 38505, 38510, 38520, 38525, 38530, 43605, 47100, 48100, 50205, 54505
**Inguinal hernia repair, laparoscopic**<br>49650, 49651
**Inguinal hernia repair, open**<br>49491, 49492, 49495, 49496, 49500, 49501, 49505, 49507, 49520, 49521, 49525
**Lumbar decompression surgery**

22220, 22222, 22224, 22226, 63001, 63003, 63005, 63011, 63012, 63015, 63016, 63017, 63020, 63030, 63035, 63040, 63042, 63043, 63044, 63045, 63046, 63047, 63048, 63050, 63051, 63057, 63066, 63075, 63076, 63077, 63078, 63101, 63102, 63103, 63170, 63172, 63173, 63180, 63182, 63185, 63190, 63191, 63194, 63195, 63196, 63197, 63198, 63199, 63200, 63250, 63251, 63252, 63265, 63266, 63267, 63268, 63270, 63271, 63272, 63273, 63275, 63276, 63277, 63278, 63280, 63281, 63282, 63283, 63285, 63286, 63287, 63290, 63655, 63662, 63664, 63709
**Lumpectomy/partial mastectomy**<br><br>19120, 19125, 19126, 19301, 19302
**Simple mastectomy**<br><br>19300, 19303, 19304
**Parathyroid or thyroid surgery**<br><br>60200, 60210, 60212, 60220, 60225, 60240, 60252, 60254, 60260, 60270, 60271
**Sinus surgery**<br><br>31237, 31238, 31239, 31240, 31241, 31253, 31254, 31255, 31256, 31257, 31259, 31267, 31276, 31287, 31288, 31290, 31291, 31292, 31293, 31294, 31295, 31296, 31297, 31298
**Spinal fusion surgery**<br><br>22532, 22533, 22534, 22548, 22551, 22552, 22554, 22556, 22558, 22585, 22586, 22590, 22595, 22600, 22610, 22612, 22614, 22630, 22632, 22633, 22634, 22800, 22802, 22804, 22808, 22810, 22812, 22818, 22819, 22840, 22841, 22842, 22843, 22844, 22845, 22846, 22847, 22848, 22849, 22850, 22851, 22852, 22853, 22854, 22855, 22856, 22857, 22858, 22859, 22862, 22865
**Tonsillectomy**<br><br>42820, 42821, 42825, 42826
**Total hip arthroplasty**<br><br>27090, 27091, 27125, 27130, 27132, 27134, 27137, 27138
**Total knee arthroplasty**<br><br>27437, 27438, 27440, 27441, 27442, 27443, 27445, 27446, 27447, 27486, 27487, 27488
**Vaginal delivery**<br><br>59400, 59409, 59410, 59610, 59612, 59614

© 2020 Mikosz CA et al. *JAMA Network Open*.

ICD-10 CM codes for sickle cell disease

Sickle cell disease
D570, D571, D578, D5720, D5721, D5740, D5741, D5781

ICD-10 CM codes for malignant cancer

Malignant cancer
C00, C01, C02, C03, C04, C05, C06, C07, C08, C09, C10, C11, C12, C13, C14, C15, C16, C17, C18, C19, C20, C21, C22, C23, C24, C25, C26, C30, C31, C32, C33, C34, C37, C38, C39, C40, C41, C43, C45, C46, C47, C48, C49, C4A, C50, C51, C52, C53, C54, C55, C56, C57, C58, C60, C61, C62, C63, C64, C65, C66, C67, C68, C69, C70, C71, C72, C73, C74, C75, C76, C77, C78, C79, C7A, C7B, C80, C81, C82, C83, C84, C85, C86, C88, C90, C91, C92, C93, C94, C95, C96, D03, D37, D38, D39, D40, D41, D42, D43, D44, D45, D46, D47, D48, D49, G893

© 2020 Mikosz CA et al. *JAMA Network Open*.

eTable 1. Summary Statistics of Privately Insured Population by Pain Indication, 2017.[a]

	All eligibles[b]	With any postsurgical pain indication		With any nonsurgical acute pain indication		With any chronic pain indication		With sickle cell disease		With cancer	
	Total (%)	Total (%)	With linked opioid rx (%)	Total (%)	With linked opioid rx (%)	Total (%)	With linked opioid rx (%)	Total (%)	With linked opioid rx (%)	Total (%)	With linked opioid rx (%)
**Total**	18,016,259 (100)	354,672 (100)	239,197 (100)	1,313,709 (100)	211,430 (100)	1,474,731 (100)	538,963 (100)	1,192 (100)	710 (100)	146,436 (100)	64,403 (100)
**Gender**											
Male	8,958,649 (49.7)	119,359 (33.7)	89,559 (37.4)	559,983 (42.6)	95,472 (45.2)	563,992 (38.2)	205,793 (38.2)	491 (41.2)	305 (43.0)	62,446 (42.6)	27,484 (42.7)
Female	9,057,610 (50.3)	235,313 (66.3)	149,638 (62.6)	753,726 (57.4)	115,958 (54.8)	910,739 (61.8)	333,170 (61.8)	701 (58.8)	405 (57.0)	83,990 (57.4)	36,919 (57.3)
**Age Groups**											
0-18	3,299,508 (18.3)	18,470 (5.2)	8,382 (3.5)	143,959 (11.0)	7,026 (3.3)	51,862 (3.5)	3,924 (0.7)	253 (21.2)	73 (10.3)	1,060 (0.7)	314 (0.5)
19-64	11,413,433 (63.4)	233,370 (65.8)	159,219 (66.6)	808,799 (61.6)	141,415 (66.9)	796,623 (54.0)	292,798 (54.3)	825 (69.6)	577 (81.3)	45,809 (31.3)	24,355 (37.8)
65+	3,303,318 (18.3)	102,832 (29.0)	71,596 (29.9)	360,951 (27.5)	62,989 (29.8)	626,246 (42.5)	242,241 (44.9)	114 (9.2)	60 (8.5)	99,567 (68.0)	39,734 (61.7)

[a] Data from the OptumLabs Data Warehouse, 2017. Data described here reflects patient/enrollee counts, not visit/procedure counts.
[b] "All eligibles" are those patients with both medical and prescription drug coverage during the study period.

© 2020 Mikosz CA et al. *JAMA Network Open*.

eTable 2. Summary Statistics of Medicaid Population by Pain Indication, Q4 2016 to Q3 2017[a]

	All eligibles[b]	With any postsurgical pain indication		With any nonsurgical acute pain indication		With any chronic pain indication		With sickle cell disease		With cancer	
	Total (%)	Total (%)	With linked opioid rx (%)	Total (%)	With linked opioid rx (%)	Total (%)	With linked opioid rx (%)	Total (%)	With linked opioid rx (%)	Total (%)	With linked opioid rx (%)
**Total**	11,453,392 (100)	278,812 (100)	153,018 (100)	1,004,374 (100)	156,577 (100)	513,131 (100)	250,181 (100)	6,925 (100)	4,132 (100)	17,064 (100)	11,968 (100)
**Gender**											
Male	5,023,626 (43.9)	44,895 (16.1)	25,790 (16.9)	376,030 (37.4)	47,768 (30.5)	165,146 (32.2)	79,860 (31.9)	3,349 (48.4)	1,966 (47.6)	6,913 (40.5)	4,873 (40.7)
Female	6,429,766 (56.1)	233,917 (83.9)	127,228 (83.1)	628,344 (62.6)	108,809 (69.5)	347,985 (67.8)	170,321 (68.1)	3,576 (51.6)	2,166 (52.4)	10,151 (59.5)	7,095 (59.3)
**Age Groups**											
0-18	6,544,487 (57.1)	58,635 (21.0)	24,554 (16.0)	467,814 (46.6)	18,545 (11.8)	91,061 (17.7)	9,856 (3.9)	4,271 (61.7)	1,942 (47.0)	2,131 (12.5)	903 (7.5)
19-64	4,840,494 (42.3)	218,907 (78.5)	127,572 (83.4)	530,302 (52.8)	136,629 (87.3)	412,224 (80.3)	234,920 (93.9)	2,646 (38.2)	2,185 (52.9)	14,132 (82.8)	10,526 (88.0)
65+	68,411 (0.6)	1,270 (0.5)	892 (0.6)	6,258 (0.6)	1,403 (0.9)	9,846 (1.9)	5,405 (2.2)	8 (0.1)	5 (0.1)	801 (4.7)	539 (4.5)

[a] Data from the MarketScan Multi-State Medicaid Database, Q4 2016 to Q3 2017. Data described here reflects patient/enrollee counts, not visit/procedure counts.
[b] "All eligibles" are those patients with both medical and prescription drug coverage during the study period.

© 2020 Mikosz CA et al. *JAMA Network Open*.

eTable 3. Opioid Prescribing Rates and Amounts[a] for Postsurgical Pain Management Among Patients on Long-term Opioid Therapy[b] in the United States, by Indication and Insurance Type, 2017

	Privately insured[c]						Medicaid[d]					
			Days' Supply, in Days		Daily Dosage, in MME				Days' Supply, in Days		Daily Dosage, in MME	
Procedure	# Procedures with rx	% Procedures with Rx (95% CI)	Mean (95% CI)	Median (Q1, Q3)	Mean (95% CI)	Median (Q1, Q3)	# Procedures with rx	% Procedures with Rx (95% CI)	Mean (95% CI)	Median (Q1, Q3)	Mean (95% CI)	Median (Q1, Q3)
**Vaginal delivery**	57	≥81[e]	8.3 (6.8-9.8)	7.0 (5.0, 9.0)	41.2 (35.5-46.9)	37.5 (20.0, 56.3)	273	94.8 (92.2-97.4)	9.0 (8.2-9.8)	7.0 (5.0, 10.0)	43.8 (40.5-47.2)	33.8 (22.5, 56.3)
**Total knee arthroplasty**	3,652	96.7 (96.1-97.3)	10.8 (10.5-11.0)	8.0 (6.0, 12.0)	69.9 (68.2-71.5)	60.0 (37.5, 90.0)	1,252	98.7 (98.0-99.3)	10.1 (9.7-10.4)	8.0 (5.0, 12.0)	68.8 (66.4-71.3)	60.0 (33.3, 90.0)
**Sinus surgery**	1,237	95.3 (94.1-96.5)	14.6 (13.6-15.7)	7.0 (4.0, 30.0)	50.8 (47.3-54.2)	40.0 (28.8, 60.0)	727	98.8 (98.0-99.6)	13.4 (12.4-14.5)	7.0 (5.0, 30.0)	49.3 (45.7-53.0)	40.0 (27.0, 60.0)
**Cholecystectomy, laparoscopic**	1,541	97.2 (96.3-98.0)	9.6 (9.1-10.1)	5.0 (4.0, 10.0)	50.3 (48.4-52.3)	45.0 (30.0, 60.0)	1,142	99.3 (98.8-99.8)	8.6 (8.1-9.0)	5.0 (3.0, 9.0)	47.2 (45.6-48.8)	42.3 (30.0, 60.0)
**Cesarean section**	57	≥81[e]	9.2 (7.6-10.8)	7.0 (5.0, 10.0)	55.3 (48.5-62.1)	45.0 (30.0, 75.0)	263	97.8 (96.0-99.5)	8.0 (7.3-8.7)	7.0 (5.0, 8.0)	50.2 (47.1-53.3)	45.0 (30.0, 60.0)
**Total hip arthroplasty**	2,324	95.2 (94.3-96.0)	10.3 (10.0-10.6)	8.0 (5.0, 10.0)	67.5 (65.7-69.2)	60.0 (37.5, 90.0)	857	98.4 (97.6-99.2)	10.5 (10.1-11.0)	8.0 (6.0, 12.0)	67.8 (65.0-70.6)	57.1 (32.1, 90.0)
**Lumpectomy/ partial mastectomy**	300	95.2 (92.9-97.6)	10.9 (9.4-12.5)	5.0 (3.0, 15.0)	46.3 (41.4-51.2)	37.5 (25.0, 56.3)	224	99.6 (98.7-100.0)	9.5 (8.2-10.9)	5.0 (3.0, 10.0)	45.6 (39.8-51.4)	37.5 (22.5, 56.3)
**Combined spinal fusion and lumbar decompression surgery**	3,908	96.4 (95.9-97.0)	12.9 (12.5-13.3)	10.0 (7.0, 15.0)	76.1 (73.3-78.8)	60.0 (40.0, 90.0)	1,262	98.1 (97.4-98.9)	10.5 (10.0-11.1)	8.0 (6.0, 13.0)	74.9 (70.6-79.3)	60.0 (40.0, 90.0)
**Lumbar decompression**	2,555	97.9 (97.3-98.4)	11.4 (11.1-11.7)	9.0 (6.0, 14.0)	66.9 (65.1-68.8)	60.0 (36.0, 90.0)	903	99.0 (98.4-99.7)	10.8 (10.3-11.3)	8.0 (5.0, 14.0)	65.1 (62.4-67.8)	56.3 (30.0, 90.0)
**Arthroscopic rotator cuff repair**	1,169	98.8 (98.2-99.4)	9.7	7.0	66.5	60.0	608	99.7 (99.2-100.0)	9.6	7.0	62.6	56.3

© 2020 Mikosz CA et al. *JAMA Network Open*.

Procedure	Privately insured[c]						Medicaid[d]					
	# Procedures with rx	% Procedures with Rx (95% CI)	Days' Supply, in Days		Daily Dosage, in MME		# Procedures with rx	% Procedures with Rx (95% CI)	Days' Supply, in Days		Daily Dosage, in MME	
			Mean (95% CI)	Median (Q1, Q3)	Mean (95% CI)	Median (Q1, Q3)			Mean (95% CI)	Median (Q1, Q3)	Mean (95% CI)	Median (Q1, Q3)
			(9.3-10.0)	(5.0, 10.0)	(64.4-68.7)	(40.0, 90.0)			(9.1-10.1)	(5.0, 10.0)	(60.0-65.2)	(33.8, 90.0)
**Spinal fusion**	2,682	97.1 (96.5-97.8)	12.4 (12.1-12.7)	10.0 (7.0, 15.0)	73.9 (72.0-75.8)	60.0 (40.0, 90.0)	1,387	98.6 (98.0-99.2)	11.3 (10.9-11.7)	10.0 (7.0, 14.0)	71.0 (68.6-73.5)	60.0 (37.5, 90.0)
**Tonsillectomy**	71	≥84[e]	12.0 (9.8-14.2)	7.0 (5.0, 17.0)	55.2 (48.0-62.4)	43.9 (30.0, 75.0)	88	100.0	8.1 (6.6-9.7)	6.0 (4.0, 8.0)	57.9 (49.9-65.9)	46.9 (32.1, 70.3)
**Abdominal solid organ resection, laparoscopic**	383	≥97[e]	8.6 (7.9-9.4)	5.0 (4.0, 8.0)	54.6 (51.5-57.8)	45.0 (30.0, 75.0)	415	99.8 (99.3-100.0)	8.2 (7.5-8.9)	5.0 (4.0, 8.0)	49.2 (46.5-52.0)	42.9 (30.0, 60.0)
**Appendectomy, laparoscopic**	278	94.6 (92.0-97.2)	9.7 (8.4-11.0)	5.0 (4.0, 10.0)	54.8 (49.2-60.3)	45.0 (30.0, 60.0)	206	97.6 (95.6-99.7)	8.7 (7.5-9.8)	5.0 (4.0, 10.0)	48.6 (43.0-54.2)	45.0 (30.0, 60.0)
**Inguinal hernia repair, open**	374	≥97[e]	10.3 (9.2-11.4)	6.0 (4.0, 10.0)	47.8 (44.6-51.0)	41.7 (30.0, 60.0)	181	98.4 (96.5-100.0)	8.6 (7.4-9.9)	5.0 (3.0, 8.0)	57.4 (51.0-63.8)	45.0 (30.0, 75.0)
**Excisional biopsy**	279	≥96[e]	13.3 (11.4-15.2)	7.0 (5.0, 30.0)	64.1 (55.0-73.3)	45.0 (30.0, 67.5)	190	96.4 (93.9-99.0)	10.1 (8.6-11.6)	6.0 (4.0, 10.0)	54.4 (48.6-60.1)	45.0 (30.0, 67.5)
**Coronary artery bypass**	407	89.3 (86.4-92.1)	11.8 (10.3-13.3)	8.0 (5.0, 13.0)	53.4 (46.0-60.7)	43.6 (30.0, 60.0)	166	93.3 (89.6-96.9)	11.5 (9.3-13.6)	9.0 (5.0, 13.0)	55.1 (44.8-65.5)	45.0 (30.0, 62.5)
**Inguinal hernia repair, laparoscopic**	217	≥95[e]	8.8 (7.6-10.0)	5.0 (4.0, 8.0)	55.0 (50.4-59.5)	45.0 (30.0, 71.3)	85	100.0	9.4 (7.5-11.3)	6.0 (4.0, 10.0)	49.0 (43.8-54.3)	47.5 (30.0, 65.3)
**Simple mastectomy**	106	≥89[e]	10.3 (6.9-13.7)	5.0 (4.0, 10.0)	58.8 (49.6-68.0)	45.0 (32.1, 75.0)	44	100.0	11.8 (8.1-15.4)	7.0 (4.0, 15.0)	55.6 (41.3-70.0)	43.9 (30.0, 62.5)
**Arthroscopic knee surgery**	104	≥89[e]	9.5 (8.2-10.8)	7.0 (5.0, 10.0)	57.2 (52.6-61.9)	46.9 (37.5, 75.0)	120	100.0	10.1 (8.9-11.3)	8.0 (5.0, 10.0)	62.9 (55.3-70.4)	59.3 (40.0, 80.0)

© 2020 Mikosz CA et al. *JAMA Network Open*.

	Privately insured[c]						Medicaid[d]					
			Days' Supply, in Days		Daily Dosage, in MME				Days' Supply, in Days		Daily Dosage, in MME	
**Procedure**	**# Procedures with rx**	**% Procedures with Rx (95% CI)**	**Mean (95% CI)**	**Median (Q1, Q3)**	**Mean (95% CI)**	**Median (Q1, Q3)**	**# Procedures with rx**	**% Procedures with Rx (95% CI)**	**Mean (95% CI)**	**Median (Q1, Q3)**	**Mean (95% CI)**	**Median (Q1, Q3)**
**Colectomy, laparoscopic**	171	≥93[e]	9.1 (7.4-10.9)	6.5 (4.0, 10.0)	59.8 (50.3-69.4)	45.0 (30.0, 75.0)	69	98.6 (95.8-100.0)	9.5 (6.8-12.1)	6.0 (5.0, 10.0)	48.3 (39.1-57.5)	43.9 (28.9, 60.0)
**Parathyroid or thyroid surgery**	205	≥94[e]	9.3 (7.3-11.2)	5 (4, 8)	47.2 (42.0-52.4)	40.0 (30.0, 60.0)	162	97.6 (95.3-99.9)	10.0 (8.4-11.6)	5.0 (3.0, 10.0)	51.0 (45.5-56.5)	45.0 (28.1, 60.0)
**Cholecystectomy, open**	94	87.9 (81.7-94.0)	9.5 (6.0-13.1)	5 (4, 8)	42.2 (30.7-53.6)	33.3 (27.0, 48.0)	66	91.7 (85.3-98.1)	9.4 (6.4-12.5)	5.0 (4.0, 10.0)	55.3 (43.3-67.3)	45.0 (30.0, 60.0)
**Colectomy, open**	228	90.8 (87.3-94.4)	10.1 (7.6-12.5)	7 (4, 10)	69.4 (48.7-90.1)	45.0 (30.0, 75.0)	148	95.5 (92.2-98.8)	9.7 (8.0-11.4)	7.0 (5.0, 10.0)	76.6 (48.6-104.5)	45.0 (28.0, 75.0)

[a] Reported outcome data (prescribing rate, MME, and days' supply) reflect prescriptions supplied for a specific procedure or visit, meaning that the prescribing rate is anchored to visits/procedures.
[b] Long-term opioid therapy (LTOT) is defined as 1) having at least three opioid prescriptions in the three months prior to the index visit; 2) more than 60 total days of opioid supply; and 3) the gap between the end of one prescription and the next prescription was fewer than 10 days.
[c] Data from the OptumLabs Data Warehouse, 2017.
[d] Data from the Marketscan Multi-State Medicaid Database, Q4 2016—Q3 2017.
[e] Due to the small-cell suppression policy of OLDW, this is the largest allowable percentage we can report.

Abbreviations: LTOT = long-term opioid therapy; Rx = prescription; MME = morphine milligram equivalents (available at https://www.cdc.gov/drugoverdose/resources/data.html). (Q1, Q3) represent the interquartile range.

© 2020 Mikosz CA et al. *JAMA Network Open*.

eTable 4. Opioid Prescribing Rates and Amounts[a] for Nonsurgical Acute Pain Among Privately Insured Patients Not on Long-term Opioid Therapy[b] in the United States, by Indication and Age Group, 2017[c]

New Indication	Age in years	# Visits with rx (%)	Days Supply, in Days		Daily Dosage, in MME	
			Mean (95% CI)	Median (Q1, Q3)	Mean (95% CI)	Median (Q1, Q3)
Abdominal pain	0-18	1,244 (2.9)	4.0 (3.8-4.2)	3.0 (2.0,5.0)	27.9 (26.7-29.0)	25.0 (18.8,33.3)
	19-64	31,617 (13.6)	5.3 (5.2-5.3)	3.0 (3.0,5.0)	32.2 (31.9-32.4)	30.0 (20.0,37.5)
	65+	10,041 (10.3)	7.7 (7.6-7.9)	5.0 (3.0,8.0)	29.3 (28.9-29.7)	25.0 (18.8,35.0)
Acute low back pain	0-18	376 (1.6)	5.4 (4.9-5.9)	4.0 (3.0,6.0)	27.2 (25.8-28.7)	24.5 (18.0,32.7)
	19-64	52,273 (13.5)	10.6 (10.5-10.7)	7.0 (3.0,15.0)	29.0 (28.8-29.2)	22.5 (18.0,35.0)
	65+	36,150 (15.2)	13.5 (13.3-13.6)	10.0 (5.0,21.0)	25.8 (25.6-26.0)	20.0 (15.0,30.0)
Acute migraine	0-18	139 (0.9)	5.7 (4.8-6.7)	5.0 (3.0,6.0)	26.9 (23.9-30.0)	25.0 (15.0,33.3)
	19-64	6,783 (4.9)	11.8 (11.6-12.1)	7.0 (4.0,15.0)	29.5 (28.9-30.2)	24.0 (16.7,37.5)
	65+	1,299 (5.4)	17.0 (16.2-17.7)	15.0 (5.0,30.0)	27.3 (26.0-28.6)	20.0 (15.0,33.3)
Dental pain	0-18	3,719 (13.6)	3.4 (3.3-3.4)	3.0 (2.0, 4.0)	36.8 (36.3-37.3)	33.3 (25.0, 50.0)
	19-64	13,434 (39.8)	4.0 (4.0-4.1)	3.0 (2.0, 5.0)	33.9 (33.6-34.1)	30.0 (20.0, 45.0)
	65+	1,974 (21.6)	6.1 (5.8-6.5)	4.0 (3.0, 5.0)	28.5 (27.8-29.1)	25.0 (19.3, 35.0)
Rib fractures	0-18	36 (15.9)	5.7 (4.5-6.8)	5.0 (3.0, 8.0)	33.4 (27.4-39.4)	30.0 (21.3, 41.4)
	19-64	4,575 (51.8)	6.4 (6.3-6.6)	5.0 (3.0, 7.0)	36.0 (35.3-36.6)	30.0 (20.0, 45.0)
	65+	4,844 (40.2)	7.3 (7.1-7.5)	5.0 (3.0, 8.0)	29.8 (29.3-30.3)	25.0 (20.0, 37.5)
Herpes zoster	0-18	26 (3.6)	4.7 (3.4-6.0)	4.0 (3.0, 5.0)	24.9 (19.6-30.1)	20.0 (16.9, 33.3)
	19-64	6,056 (15.6)	7.4 (7.2-7.6)	5.0 (3.0, 8.0)	28.3 (27.9-28.7)	25.0 (18.8, 33.8)
	65+	5,206 (15.6)	9.6 (9.4-9.9)	7.0 (4.0, 10.0)	26.1 (25.7-26.5)	21.4 (16.7, 30.0)
Renal colic	0-18	267 (19.4)	3.5 (3.3-3.7)	3.0 (2.0, 4.0)	32.1 (29.8-34.4)	29.1 (20.0, 37.5)
	19-64	20,841 (25.0)	4.8 (4.8-4.9)	3.0 (3.0, 5.0)	36.0 (35.7-36.3)	30.0 (21.4, 45.0)
	65+	6,777 (12.6)	6.3 (6.1-6.5)	4.0 (3.0, 6.0)	34.0 (33.6-34.5)	30.0 (20.0, 45.0)
Musculoskeletal sprains and strains	0-18	2,397 (2.9)	5.2 (5.0-5.4)	5.0 (3.0, 6.0)	34.8 (33.7-35.9)	30.0 (20.0, 45.0)
	19-64	49,576 (14.3)	6.3 (6.3-6.4)	5.0 (3.0, 7.0)	33.2 (33.0-33.4)	27.5 (20.0, 40.0)
	65+	17,461 (15.9)	7.8 (7.6-7.9)	5.0 (3.0, 8.0)	28.6 (28.3-28.9)	22.5 (18.8, 33.3)

[a] Reported outcome data (prescribing rate, MME, and days' supply) reflect prescriptions supplied for a specific procedure or visit, meaning that the prescribing rate is anchored to visits/procedures.

© 2020 Mikosz CA et al. *JAMA Network Open*.

[b] Patients not on long-term opioid therapy (LTOT) were those whose prescriptions did not meet the LTOT criteria. LTOT is defined as 1) having at least three opioid prescriptions in the three months prior to the index visit; 2) more than 60 total days of opioid supply; and 3) the gap between the end of one prescription and the next prescription was fewer than 10 days.
[c] Data are from the OptumLabs Data Warehouse, 2017.

Abbreviations: Rx = prescription; MME = morphine milligram equivalents (available at https://www.cdc.gov/drugoverdose/resources/data.html). (Q1, Q3) represent the interquartile range.

© 2020 Mikosz CA et al. *JAMA Network Open*.

eTable 5. Opioid Prescribing Rates and Amounts[a] for Chronic Noncancer Pain Among Privately Insured Patients in the United States, by Indication, History of Opioid Usage, and Age Group[b,c], 2017

		19-64 YEARS		65+ YEARS	
		Not on LTOT[d]	On LTOT[e]	Not on LTOT	On LTOT
**Chronic nonradicular back pain**	# Patients with rx (%)	59,897 (17.8)	63,737 (89.7)	52,822 (24.9)	39,522 (84.5)
	Mean # rx (95% CI)	1.7 (1.7-1.7)	3.1 (3.1-3.1)	1.6 (1.6-1.6)	2.5 (2.5-2.6)
	Mean days' supply, in days (95% CI)	29.9 (29.6-30.1)	85.5 (85.0-85.9)	29.9 (29.6-30.1)	71.8 (71.3-72.2)
	Mean daily dosage, in MME (95% CI)	31.7 (31.5-31.9)	60.1 (59.6-60.5)	27.1 (26.9-27.3)	49.8 (49.4-50.3)
**Chronic radicular back pain**	# Patients with rx (%)	19,100 (28.0)	26,574 (89.2)	15,441 (29.0)	13,935 (84.4)
	Mean # rx (95% CI)	1.9 (1.9-1.9)	3.0 (3.0-3.1)	1.7 (1.6-1.7)	2.6 (2.5-2.6)
	Mean days' supply, in days (95% CI)	36.2 (35.7-36.7)	84.6 (84.0-85.3)	33.4 (33.0-33.9)	72.5 (71.7-73.3)
	Mean daily dosage, in MME (95% CI)	32.1 (31.7-32.5)	61.8 (61.2-62.5)	27.8 (27.5-28.2)	52.2 (51.4-52.9)
**Chronic neck pain**	# Patients with rx (%)	21,787 (15.0)	25,184 (88.9)	11,338 (19.4)	10,457 (83.7)
	Mean # rx (95% CI)	1.7 (1.7-1.8)	3.0 (3.0-3.1)	1.5 (1.5-1.6)	2.6 (2.5-2.6)
	Mean days' supply, in days (95% CI)	30.3 (29.8-30.7)	84.7 (84.0-85.4)	29.4 (28.9-29.9)	73.2 (72.3-74.2)
	Mean daily dosage, in MME (95% CI)	31.9 (31.5-32.2)	61.7 (61.0-62.4)	28.1 (27.7-28.5)	53.9 (53.0-54.9)
**Fibromyalgia**	# Patients with rx (%)	4,495 (23.7)	7,088 (79.5)	1,986 (23.4)	2,626 (73.9)
	Mean # rx (95% CI)	1.5 (1.5-1.5)	2.5 (2.4-2.5)	1.4 (1.3-1.4)	2.1 (2.0-2.2)
	Mean days' supply, in days (95% CI)	33.6 (32.7-34.4)	69.8 (68.5-71.0)	33.3 (32.2-34.5)	60.2 (58.5-61.8)
	Mean daily dosage, in MME (95% CI)	30.0 (29.2-30.8)	58.1 (56.8-59.4)	28.1 (27.0-29.2)	51.6 (49.8-53.4)
**Inflammatory joint disorder**	# Patients with rx (%)	46,414 (19.5)	33,820 (84.9)	42,744 (21.1)	22,852 (78.3)
	Mean # rx (95% CI)	1.6 (1.6-1.6)	2.7 (2.7-2.7)	1.4 (1.4-1.5)	2.2 (2.2-2.2)
	Mean days' supply, in days (95% CI)	22.8 (22.5-23.0)	73.7 (73.1-74.2)	23.3 (23.1-23.5)	60.8 (60.2-61.3)
	Mean daily dosage, in MME (95% CI)	34.6 (34.4-34.8)	57.9 (57.3-58.4)	28.4 (28.2-28.6)	47.1 (46.6-47.7)
**Irritable bowel syndrome**	# Patients with rx (%)	831 (6.3)	633 (65.3)	682 (7.0)	457 (60.9)
	Mean # rx (95% CI)	1.3 (1.2-1.3)	1.9 (1.8-2.0)	1.2 (1.2-1.3)	1.5 (1.5-1.6)
	Mean days' supply, in days (95% CI)	19.4 (18.1-20.7)	51.8 (49.0-54.7)	23.0 (21.6-24.4)	43.0 (40.6-45.5)
	Mean daily dosage, in MME (95% CI)	31.0 (29.3-32.7)	54.2 (50.0-58.4)	27.3 (25.8-28.7)	40.1 (36.7-43.5)

© 2020 Mikosz CA et al. *JAMA Network Open*.

		19-64 YEARS		65+ YEARS	
		Not on LTOT[d]	On LTOT[e]	Not on LTOT	On LTOT
**Non-migraine headaches**	# Patients with rx (%)	5,364 (12.3)	3,894 (77.1)	2,337 (14.1)	1,538 (70.2)
	Mean # rx (95% CI)	1.4 (1.4-1.5)	2.2 (2.2-2.3)	1.3 (1.3-1.3)	1.8 (1.8-1.9)
	Mean days' supply, in days (95% CI)	18.5 (17.9-19.1)	59.3 (57.9-60.7)	19.0 (18.3-19.8)	48.8 (47.0-50.7)
	Mean daily dosage, in MME (95% CI)	30.0 (29.3-30.6)	55.6 (53.9-57.3)	26.0 (25.2-26.7)	44.9 (42.9-47.0)
**Osteoarthritis/joint cartilage conditions**	# Patients with rx (%)	25,273 (21.8)	20,303 (80.9)	39,876 (17.3)	22,760 (74.8)
	Mean # rx (95% CI)	1.5 (1.5-1.5)	2.4 (2.4-2.4)	1.4 (1.4-1.4)	2.0 (2.0-2.0)
	Mean days' supply, in days (95% CI)	22.7 (22.4-23.0)	65.5 (64.8-66.2)	24.3 (24.1-24.5)	55.3 (54.8-55.8)
	Mean daily dosage, in MME (95% CI)	36.5 (36.2-36.9)	56.0 (55.2-56.7)	28.4 (28.2-28.6)	42.9 (42.3-43.4)
**Periarticular/soft tissue disorders**	# Patients with rx (%)	16,756 (17.4)	7,846 (78.1)	10,754 (15.5)	4,837 (70.4)
	Mean # rx (95% CI)	1.5 (1.5-1.5)	2.4 (2.3-2.4)	1.3 (1.3-1.3)	1.9 (1.9-2.0)
	Mean days' supply, in days (95% CI)	16.6 (16.3-16.9)	62.9 (61.8-63.9)	18.9 (18.5-19.3)	54.0 (52.9-55.0)
	Mean daily dosage, in MME (95% CI)	40.8 (40.4-41.2)	55.4 (54.3-56.5)	32.9 (32.5-33.4)	46.0 (44.8-47.3)

[a] Reported outcome data (prescribing rate, MME, days' supply, and number of prescriptions) is anchored to patients, reflecting all prescriptions supplied to a patient for visits related to that indication during the three months following the index diagnosis.

[b] Data from the OptumLabs Data Warehouse, 2017.

[c] Ages 0-18 years are not included here because of too little data to report.

[d] Patients not on long-term opioid therapy (LTOT) were those whose prescriptions did not meet the LTOT criteria.

[e] Long-term opioid therapy (LTOT) is defined as 1) having at least three opioid prescriptions in the three months prior to the index visit; 2) more than 60 total days of opioid supply; and 3) the gap between the end of one prescription and the next prescription was fewer than 10 days.

Abbreviations: Rx = prescription; MME = morphine milligram equivalents (available at https://www.cdc.gov/drugoverdose/resources/data.html); LTOT = long-term opioid therapy.

© 2020 Mikosz CA et al. *JAMA Network Open*.

eTable 6. Opioid Prescribing Rates and Amounts[a] for Postsurgical Pain Management Among Privately Insured Patients Not on Long-term Opioid Therapy[b] in the United States, by Indication and Age Group[c, d], 2017

Procedure	Age, in yrs	# Procedures with rx (%)	Days Supply, in Days		Daily Dosage, in MME		
			Mean (95% CI)	Median (Q1, Q3)	Mean (95% CI)	Median (Q1, Q3)	Total # of procedures
Vaginal delivery	0-18	96 (22.7)	4.2 (3.7-4.6)	4.0 (3.0, 5.0)	35.7 (32.6-38.8)	31.3 (25.0,45.0)	423
	19-64	14,070 (23.6)	4.1 (4.0-4.1)	4.0 (3.0.5.0)	39.1 (38.8-39.4)	37.5 (27.0,50.0)	59,586
Total knee arthroplasty	19-64	10,601 (88.9)	8.3 (8.3-8.4)	7.0 (5.0,10.0)	66.4 (65.8-67.0)	60.0 (38.3,90.0)	11,924
	65+	16,925 (74.6)	8.7 (8.6-8.7)	8.0 (5.0,10.0)	58.9 (58.5-59.3)	56.3 (37.5,83.3)	22,698
Sinus surgery	0-18	887 (45.2)	5.0 (4.7-5.2)	5.0 (3.0,6.0)	36.7 (35.1-38.3)	33.3 (25.0,50.0)	1,962
	19-64	16,776 (69.2)	4.8 (4.8-4.9)	4.0 (3.0,5.0)	43.2 (42.8-43.6)	40.0 (30.0,50.0)	24,236
	65+	3,998 (50.5)	5.0 (4.8-5.1)	4.0 (3.0,5.0)	39.2 (38.4-40.0)	36.0 (25.0,50.0)	7,922
Cholecystectomy, laparoscopic	0-18	300 (75.2)	4.3 (4.1-4.6)	4.0 (3.0,5.0)	38.0 (36.0-39.9)	32.1 (25.0,50.0)	399
	19-64	16,982 (85.5)	4.7 (4.7-4.7)	4.0 (3.0,5.0)	42.9 (42.6-43.2)	37.5 (30.0,50.0)	19,871
	65+	6,382 (69.0)	4.8 (4.8-4.9)	4.0 (3.0,5.0)	39.8 (39.3-40.2)	37.5 (25.0,50.0)	9,247
Cesarean section	0-18	67 (76.1)	5.1 (4.5-5.8)	5.0 (3.0,5.0)	43.0 (38.5-47.5)	45.0 (30.0,56.3)	88
	19-64	24,178 (78.0)	4.9 (4.9-5.0)	5.0 (3.0,5.0)	48.5 (48.2-48.7)	45.0 (32.0,60.0)	30,978
Total hip arthroplasty	19-64	6,280 (88.9)	8.3 (8.2-8.4)	7.0 (5.0,10.0)	62.0 (61.2-62.7)	56.3 (37.5,85.7)	7,066
	65+	8,088 (68.9)	8.5 (8.4-8.6)	8.0 (5.0,10.0)	53.8 (53.2-54.3)	45.7 (30.0,75.0)	11,731
Lumpectomy/ partial mastectomy	0-18	105 (60.3)	3.9 (3.3-4.5)	3.0 (2.0,5.0)	32.4 (30.0-34.8)	30.0 (25.0,40.0)	174
	19-64	3,938 (72.0)	4.2 (4.1-4.3)	4.0 (3.0,5.0)	38.1 (37.5-38.6)	33.3 (25.0,50.0)	5,471
	65+	2,312 (66.9)	4.4 (4.2-4.5)	4.0 (3.0,5.0)	36.4 (35.7-37.1)	33.3 (25.0,45.0)	3,454
Combined spinal fusion and lumbar decompression surgery	0-18	34 (53.1)	7.6 (5.5-9.7)	8.0 (4.0,10.0)	60.9 (37.9-83.8)	45.0 (31.3,90.0)	64
	19-64	5,351 (83.4)	9.6 (9.4-9.8)	8.0 (6.0,10.0)	68.2 (66.8-69.6)	60.0 (38.5,90.0)	6,414
	65+	5,235 (69.0)	9.4 (9.2-9.6)	8.0 (5.0,10.0)	57.9 (56.8-59.1)	50.0 (30.0,83.3)	7,587
Lumbar decompression	0-18	83 (54.2)	5.8 (5.1-6.6)	5.0 (3.0,7.0)	37.6 (32.0-43.2)	31.3 (15.6,46.9)	153
	19-64	7,170 (90.7)	8.0 (7.9-8.1)	7.0 (5.0,10.0)	55.6 (55.0-56.3)	50.0 (30.7,75.0)	7,902
	65+	4,591 (80.3)	8.3 (8.2-8.4)	7.0 (5.0,10.0)	51.2 (50.5-52.0)	45.0 (30.0,63.0)	5,715

© 2020 Mikosz CA et al. *JAMA Network Open*.

Procedure	Age, in yrs	# Procedures with rx (%)	Days Supply, in Days		Daily Dosage, in MME		
			Mean (95% CI)	Median (Q1, Q3)	Mean (95% CI)	Median (Q1, Q3)	Total # of procedures
**Arthroscopic rotator cuff repair**	0-18	21 (63.6)	5.6 (4.8-6.4)	5.0 (4.0,7.0)	62.4 (52.7-72.0)	60.0 (45.0,75.0)	33
	19-64	7,731 (93.3)	6.5 (6.5-6.6)	5.0 (5.0,8.0)	63.6 (63.0-64.2)	60.0 (40.0,84.4)	8,287
	65+	4,786 (92.8)	6.8 (6.7-6.9)	6.0 (5.0,8.0)	60.9 (60.2-61.7)	60.0 (37.5,80.0)	5,158
**Spinal fusion**	0-18	266 (68.2)	9.1 (8.5-9.7)	8.0 (5.0,10.0)	49.2 (45.7-52.6)	45.0 (30.0,60.0)	390
	19-64	6,301 (87.0)	9.0 (8.8-9.1)	8.0 (5.0,10.0)	60.4 (59.6-61.1)	56.3 (33.3,80.4)	7,244
	65+	2,639 (71.5)	9.0 (8.8-9.2)	8.0 (5.0,10.0)	53.9 (52.8-54.9)	45.0 (30.0,67.5)	3,693
**Tonsillectomy**	0-18	3,134 (32.8)	7.8 (7.6-7.9)	7.0 (5.0,10.0)	27.3 (26.6-28.1)	20.0 (12.5,35.0)	9,559
	19-64	3,043 (68.1)	6.8 (6.5-7.1)	6.0 (5.0,8.0)	55.0 (54.1-56.0)	48.0 (31.3,75.0)	4,470
	65+	52 (66.7)	7.0 (5.5-8.5)	5.0 (5.0,8.0)	47.5 (41.2-53.8)	44.1 (31.3,60.0)	78
**Abdominal solid organ resection, laparoscopic**	19-64	9,217 (87.5)	5.0 (4.9-5.0)	5.0 (3.0,6.0)	46.4 (46.0-46.9)	45.0 (30.0,56.3)	10,538
	65+	979 (73.6)	5.2 (5.0-5.3)	5.0 (3.0,6.0)	43.2 (41.9-44.5)	40.0 (30.0,50.0)	1,331
**Appendectomy, laparoscopic**	0-18	1,082 (48.9)	3.8 (3.7-3.9)	3.0 (2.0,5.0)	33.6 (32.5-34.7)	30.0 (20.0,42.0)	2,213
	19-64	6,662 (76.9)	4.6 (4.6-4.7)	4.0 (3.0,5.0)	43.9 (43.4-44.4)	40.0 (30.0,50.0)	8,666
	65+	905 (54.4)	4.9 (4.7-5.1)	4.0 (3.0,5.0)	41.2 (39.9-42.5)	37.5 (25.9,50.0)	1,663
**Inguinal hernia repair, open**	0-18	418 (39.2)	4.2 (3.9-4.5)	3.0 (2.0,5.0)	18.9 (17.3-20.5)	12.0 (7.2,25.0)	1,065
	19-64	3,864 (86.0)	4.9 (4.8-5.0)	5.0 (3.0,5.0)	46.6 (46.0-47.3)	45.0 (30.0,56.3)	4,492
	65+	3,968 (79.1)	4.9 (4.8-5.0)	5.0 (3.0,5.0)	41.8 (41.2-42.4)	37.5 (30.0,50.0)	5,019
**Excisional biopsy**	0-18	74 (33.0)	5.3 (4.6-6.0)	5.0 (3.0,7.0)	26.9 (23.8-29.9)	25.0 (18.0,33.3)	224
	19-64	1,400 (53.3)	5.1 (4.9-5.2)	5.0 (3.0,7.0)	42.9 (41.2-44.5)	37.5 (28.1,50.0)	2,625
	65+	674 (42.6)	5.1 (4.8-5.4)	5.0 (3.0,7.0)	39.0 (37.6-40.5)	33.3 (25.0,50.0)	1,584
**Coronary artery bypass**	19-64	1,266 (49.3)	7.2 (7.0-7.4)	7.0 (5.0,9.0)	47.1 (45.8-48.4)	42.9 (30.0,60.0)	2,570
	65+	1,563 (28.1)	7.1 (6.9-7.3)	7.0 (5.0,9.0)	41.6 (40.6-42.6)	37.5 (28.1,50.0)	5,558
**Inguinal hernia repair, laparoscopic**	0-18	57 (45.2)	4.2 (3.6-4.9)	4.0 (2.0,5.0)	32.9 (26.8-39.0)	25.0 (13.5,50.0)	126
	19-64	4,154 (86.6)	4.7 (4.7-4.8)	4.0 (3.0,5.0)	46.5 (45.9-47.1)	45.0 (30.0,56.3)	4,797
	65+	2,403 (79.0)	4.9 (4.7-5.0)	4.0 (3.0,5.0)	43.6 (42.8-44.3)	41.7 (30.0,50.0)	3,041

© 2020 Mikosz CA et al. *JAMA Network Open*.

Procedure	Age, in yrs	# Procedures with rx (%)	Days Supply, in Days		Daily Dosage, in MME		
			Mean (95% CI)	Median (Q1, Q3)	Mean (95% CI)	Median (Q1, Q3)	Total # of procedures
**Simple mastectomy**	0-18	28 (71.8)	4.9 (3.7-6.1)	4.0 (4.0-5.0)	46.9 (38.1-55.6)	43.9 (27.0,75.0)	39
	19-64	1,411 (88.2)	5.5 (5.3-5.6)	5.0 (4.0,7.0)	54.2 (52.7-55.8)	50.0 (32.1,75.0)	1,599
	65+	657 (74.9)	5.5 (5.2-5.9)	5.0 (3.0,6.0)	42.9 (40.8-44.9)	37.5 (30.0,50.0)	877
**Arthroscopic knee surgery**	0-18	1,684 (91.9)	6.1 (6.0-6.2)	5.0 (4.0,7.0)	57.4 (56.2-58.6)	50.0 (37.5,75.0)	1,832
	19-64	5,106 (93.4)	6.3 (6.2-6.3)	5.0 (4.0,8.0)	62.2 (61.4-62.9)	60.0 (40.0,80.0)	5,465
	65+	97 (82.9)	6.6 (6.0-7.2)	5.0 (4.0,8.0)	52.5 (47.6-57.4)	46.9 (32.1,60.0)	117
**Colectomy, laparoscopic**	19-64	1,435 (71.3)	5.3 (5.1-5.4)	5.0 (3.0,7.0)	48.1 (46.9-49.3)	45.0 (30.0,60.0)	2,014
	65+	865 (48.5)	5.4 (5.2-5.6)	5.0 (4.0,7.0)	42.3 (40.9-43.6)	40.0 (28.6,50.0)	1,783
**Colectomy, open**	19-64	654 (45.0)	5.8 (5.6-6.1)	5.0 (4.0,7.0)	45.7 (43.8-47.7)	40.0 (30.0,56.3)	1,453
	65+	532 (27.4)	5.5 (5.2-5.7)	5.0 (4.0,7.0)	40.6 (38.8-42.4)	37.5 (25.0,50.0)	1,941
**Parathyroid or thyroid surgery**	0-18	32 (59.3)	5.1 (3.5-6.7)	4.5 (3.0,5.0)	33.2 (29.3-37.2)	33.3 (25.0,40.0)	54
	19-64	2,187 (75.7)	4.7 (4.6-4.8)	4.0 (3.0,5.0)	42.4 (41.6-43.2)	38.5 (30.0,50.0)	2,890
	65+	838 (61.9)	4.9 (4.7-5.1)	4.0 (3.0,5.0)	40.3 (39.1-41.5)	37.5 (28.1,50.0)	1,353
**Cholecystectomy, open**	19-64	259 (61.2)	5.1 (4.7-5.5)	5.0 (3.0,6.0)	43.4 (40.9-45.9)	37.5 (30.0,54.0)	423
	65+	249 (42.6)	5.7 (5.2-6.1)	5.0 (4.0,7.0)	39.0 (36.7-41.3)	33.5 (26.3,48.2)	585

[a] Reported outcome data (prescribing rate, MME, and days' supply) reflect prescriptions supplied for a specific procedure or visit, meaning that the prescribing rate is anchored to visits/procedures.

[b] Patients not on long-term opioid therapy (LTOT) were those whose prescriptions did not meet the LTOT criteria. LTOT is defined as 1) having at least three opioid prescriptions in the three months prior to the index visit; 2) more than 60 total days of opioid supply; and 3) the gap between the end of one prescription and the next prescription was fewer than 10 days.

[c]Data are from the OptumLabs Data Warehouse, 2017.

[d]Not all age groups are well-represented for every procedure and are thus not listed here.

Abbreviations: Rx = prescription; MME = morphine milligram equivalents (available at https://www.cdc.gov/drugoverdose/resources/data.html). (Q1, Q3) represent the interquartile range.

© 2020 Mikosz CA et al. *JAMA Network Open*.

eTable 7. Opioid Prescribing Rates and Amounts[a] for Nonsurgical Acute Pain Among Medicaid Enrollees Not on Long-term Opioid Therapy[b] in the United States, By Age Group, Q4 2016–Q3 2017[c]

Diagnosis	Age in Years	# Procedures with rx (%)	Days Supply, in Days		Daily Dosage, in MME	
			Mean (95% CI)	Median (Q1, Q3)	Mean (95% CI)	Median (Q1, Q3)
**Abdominal pain**	0-18	4,442 (3.3)	3.9 (3.8-4.0)	3.0 (2.0, 5.0)	25.5 (25.0-26.0)	21.4 (17.5, 30.0)
	19-64	37,228 (20.2)	4.9 (4.8-4.9)	3.0 (2.0, 5.0)	30.6 (30.4-30.7)	25.0 (20.0, 37.5)
	65+	266 (12.2)	6.5 (5.6-7.5)	4.0 (3.0, 6.0)	28.7 (26.4-31.0)	25.0 (20.0, 33.3)
**Acute low back pain**	0-18	2,018 (3.4)	4.8 (4.6-5.0)	3.0 (2.0, 5.0)	25.0 (24.4-25.7)	20.0 (16.7, 30.0)
	19-64	58,254 (22.6)	10.0 (9.9-10.1)	5.0 (3.0, 15.0)	28.3 (28.1-28.5)	22.5 (17.5, 33.3)
	65+	945 (22.7)	13.0 (12.3-13.7)	8.0 (4.0, 28.0)	27.9 (25.8-29.9)	20.0 (15.0, 30.0)
**Acute migraine**	0-18	665 (1.3)	5.7 (5.2-6.1)	4.0 (3.0, 6.0)	24.5 (23.2-25.7)	20.0 (15.0, 30.0)
	19-64	9,079 (9.2)	10.1 (9.9-10.3)	5.0 (3.0, 15.0)	28.8 (28.4-29.2)	22.5 (16.7, 35.0)
	65+	53 (11.7)	12.8 (9.6-16.0)	7.0 (4.0, 30.0)	27.4 (22.6-32.2)	22.5 (15.0, 38.6)
**Dental pain**	0-18	8,196 (3.4)	4.1 (4.0-4.1)	4.0 (3.0, 5.0)	27.1 (26.7-27.5)	25.0 (12.0, 36.0)
	19-64	32,183 (32.7)	4.0 (3.9-4.0)	3.0 (2.0, 4.0)	28.2 (28.1-28.4)	25.0 (20.0, 33.3)
	65+	134 (11.8)	5.3 (4.3-6.3)	3.0 (2.0, 5.0)	26.8 (24.7-28.9)	25.0 (20.0, 33.3)
**Rib fractures**	0-18	178 (29.0)	4.7 (4.2-5.3)	4.0 (3.0, 5.0)	30.1 (27.1-33.0)	27.0 (18.0, 36.0)
	19-64	4,221 (58.9)	5.7 (5.5-5.9)	4.0 (3.0, 6.0)	32.7 (32.1-33.2)	30.0 (20.0, 37.5)
	65+	85 (47.0)	5.3 (4.4-6.2)	4.0 (3.0, 7.0)	29.4 (26.0-32.7)	27.0 (20.0, 37.5)
**Herpes zoster**	0-18	85 (6.4)	4.4 (3.7-5.1)	4.0 (3.0, 5.0)	25.4 (22.0-28.8)	21.0 (16.7, 30.0)
	19-64	3,074 (28.4)	6.3 (6.0-6.5)	4.0 (3.0, 7.0)	28.5 (28.0-29.1)	25.0 (20.0, 33.3)
	65+	88 (26.7)	8.5 (7.0-10.1)	6.0 (4.0, 10.0)	24.0 (20.9-27.2)	20.0 (15.0, 30.0)
**Renal colic**	0-18	974 (23.3)	3.9 (3.7-4.1)	3.0 (2.0, 5.0)	28.2 (27.3-29.2)	25.0 (18.8, 33.3)
	19-64	15,542 (35.2)	4.7 (4.6-4.7)	3.0 (2.0, 5.0)	33.6 (33.3-34.0)	30.0 (20.0, 40.0)
	65+	102 (16.1)	6.2 (4.9-7.6)	4.0 (3.0, 7.0)	30.4 (27.6-33.3)	27.2 (20.0, 40.0)
**Musculoskeletal sprains and strains**	0-18	6,842 (3.7)	4.6 (4.5-4.7)	4.0 (2.0, 5.0)	26.8 (26.3-27.2)	21.4 (16.7, 30.0)
	19-64	55,074 (23.7)	5.2 (5.1-5.2)	3.0 (2.0, 5.0)	28.1 (28.0-28.3)	25.0 (18.8, 33.3)
	65+	438 (25.9)	6.5 (5.8-7.2)	4.0 (3.0, 7.0)	28.9 (27.2-30.7)	25.0 (19.3, 33.3)

[a] Reported outcome data (prescribing rate, MME, and days' supply) reflect prescriptions supplied for a specific procedure or visit, meaning that the prescribing rate is anchored to visits/procedures.

© 2020 Mikosz CA et al. *JAMA Network Open*.

[b] Patients not on long-term opioid therapy (LTOT) were those whose prescriptions did not meet the LTOT criteria. LTOT is defined as 1) having at least three opioid prescriptions in the three months prior to the index visit; 2) more than 60 total days of opioid supply; and 3) the gap between the end of one prescription and the next prescription was fewer than 10 days.
[c] Data are from the MarketScan Multi-State Medicaid Database, Q4 2016—Q3 2017.

Abbreviations: Rx = prescription; MME = morphine milligram equivalents (available at https://www.cdc.gov/drugoverdose/resources/data.html). (Q1, Q3) represent the interquartile range.

© 2020 Mikosz CA et al. *JAMA Network Open*.

eTable 8. Opioid Prescribing Rates and Amounts[a] for Chronic Noncancer Pain Among Medicaid Enrollees in the United States, by Indication, Timing of Chronic Pain Diagnosis, History of Opioid Usage, and Age Group, Q4 2016–Q3 2017[b]

		0-18 YEARS		19-64 YEARS		65+ YEARS	
		Not on LTOT[c]	On LTOT[d]	Not on LTOT	On LTOT	Not on LTOT	On LTOT
**Chronic nonradicular back pain**	# Patients with rx (%)	1,189 (4.4)	*	58,571 (37.4)	60,708 (90.4)	820 (34.6)	1,007 (86.3)
	Mean # rx (95% CI)	1.3 (1.3-1.4)	*	1.9 (1.9-1.9)	2.8 (2.8-2.8)	1.7 (1.6-1.8)	2.6 (2.5-2.7)
	Mean days' supply, in days (95% CI)	10.0 (9.2-10.8)	*	31.1 (30.8-31.4)	74.0 (73.7-74.4)	31.7 (29.8-33.7)	70.1 (67.3-72.8)
	Mean daily dosage, in MME (95% CI)	27.0 (26.0-27.9)	*	28.7 (28.5-28.9)	47.7 (47.1-48.4)	25.5 (23.1-27.9)	44.3 (41.9-46.8)
**Chronic radicular back pain**	# Patients with rx (%)	48 (9.1)	*	15,502 (44.6)	20,525 (88.3)	177 (37.2)	278 (84.8)
	Mean # rx (95% CI)	1.3 (1.1-1.6)	*	2.0 (2.0-2.0)	2.7 (2.6-2.7)	1.8 (1.6-2.0)	2.6 (2.4-2.8)
	Mean days' supply, in days (95% CI)	11.2 (7.2-15.1)	*	36.7 (36.1-37.2)	71.8 (71.2-72.5)	33.5 (29.0-38.1)	70.3 (64.9-75.7)
	Mean daily dosage, in MME (95% CI)	23.3 (19.4-27.2)	*	28.8 (28.5-29.1)	47.5 (46.9-48.1)	25.0 (22.8-27.3)	43.3 (38.8-47.9)
**Chronic neck pain**	# Patients with rx (%)	192 (3.3)	*	16,753 (34.4)	20,168 (88.4)	158 (29.3)	263 (88.6)
	Mean # rx (95% CI)	1.2 (1.1-1.2)	*	1.9 (1.9-1.9)	2.7 (2.6-2.7)	1.8 (1.6-2.0)	2.7 (2.5-2.9)
	Mean days' supply, in days (95% CI)	9.1 (7.0-11.1)	*	32.6 (32.1-33.1)	71.1 (70.5-71.8)	31.1 (26.5-35.8)	70.8 (65.1-76.6)
	Mean daily dosage, in MME (95% CI)	24.4 (22.5-26.4)	*	28.9 (28.6-29.2)	48.5 (47.9-49.0)	27.5 (22.9-32.0)	45.9 (41.1-50.6)
**Fibromyalgia**	# Patients with rx (%)	19 (11.7)	*	4,194 (29.2)	5,812 (81.7)	26 (24.8)	63 (78.8)
	Mean # rx (95% CI)	1.6 (1.2-1.9)	*	1.7 (1.7-1.7)	2.3 (2.2-2.3)	1.6 (1.3-1.9)	2.2 (1.7-2.7)
	Mean days' supply, in days (95% CI)	17.3 (11.3-23.4)	*	30.2 (29.3-31.1)	61.8 (60.6-63.0)	33.6 (22.7-44.5)	60.0 (46.0-74.0)
	Mean daily dosage, in MME (95% CI)	29.1 (22.4-35.9)	*	27.7 (27.1-28.3)	44.2 (43.1-45.2)	21.0 (15.0-27.0)	48.1 (37.1-59.1)
**Inflammatory joint disorder**	# Patients with rx (%)	2,596 (7.1)	*	43,227 (36.6)	33,456 (85.4)	743 (29.8)	610 (80.1)
	Mean # rx (95% CI)	1.3 (1.3-1.4)	*	1.8 (1.8-1.8)	2.5 (2.4-2.5)	1.6 (1.6-1.7)	2.2 (2.1-2.3)
	Mean days' supply, in days (95% CI)	8.5 (8.1-8.9)	*	24.0 (23.7-24.3)	63.4 (62.9-63.9)	24.8 (23.2-26.4)	56.4 (53.2-59.5)
	Mean daily dosage, in MME (95% CI)	29.3 (28.5-30.1)	*	29.0 (28.8-29.2)	45.6 (45.2-46.1)	25.5 (23.8-27.1)	45.9 (36.1-55.7)

© 2020 Mikosz CA et al. *JAMA Network Open*.

		0-18 YEARS		19-64 YEARS		65+ YEARS	
		Not on LTOT[c]	On LTOT[d]	Not on LTOT	On LTOT	Not on LTOT	On LTOT
**Irritable bowel syndrome**	# Patients with rx (%)	31 (3.1)	*	911 (15.2)	624 (66.2)	*	*
	Mean # rx (95% CI)	1.2 (1.0-1.4)	*	1.4 (1.4-1.5)	1.7 (1.6-1.8)	*	*
	Mean days' supply, in days (95% CI)	6.4 (3.6-9.2)	*	17.1 (15.9-18.3)	44.2 (42.0-46.3)	*	*
	Mean daily dosage, in MME (95% CI)	26.6 (20.3-32.8)	*	29.3 (27.9-30.6)	40.3 (37.5-43.1)	*	*
**Non-migraine headaches**	# Patients with rx (%)	721 (2.8)	*	8,449 (21.0)	4,228 (74.3)	85 (17.4)	61 (67.8)
	Mean # rx (95% CI)	1.2 (1.1-1.2)	*	1.5 (1.5-1.5)	1.9 (1.9-1.9)	1.3 (1.1-1.4)	1.8 (1.5-2.1)
	Mean days' supply, in days (95% CI)	6.7 (6.1-7.4)	*	14.0 (13.6-14.4)	45.8 (44.8-46.8)	16.4 (13.2-19.5)	43.8 (36.9-50.7)
	Mean daily dosage, in MME (95% CI)	24.0 (23.0-25.1)	*	28.6 (28.1-29.0)	44.0 (42.8-45.2)	19.1 (16.9-21.4)	36.3 (28.8-43.8)
**Osteoarthritis/ joint cartilage conditions**	# Patients with rx (%)	237 (12.4)	*	17,872 (34.4)	18,680 (83.2)	675 (25.6)	660 (81.1)
	Mean # rx (95% CI)	1.3 (1.2-1.4)	*	1.8 (1.7-1.8)	2.3 (2.3-2.4)	1.5 (1.4-1.6)	2.1 (2.0-2.2)
	Mean days' supply, in days (95% CI)	9.4 (8.3-10.4)	*	27.4 (27.0-27.8)	61.1 (60.5-61.7)	26.3 (24.6-28.0)	54.3 (51.7-56.9)
	Mean daily dosage, in MME (95% CI)	41.4 (37.8-45.0)	*	30.3 (30.0-30.6)	44.6 (44.1-45.2)	24.6 (23.2-25.9)	41.3 (32.0-50.5)
**Periarticular/soft tissue disorders**	# Patients with rx (%)	381 (10.1)	*	9,288 (30.7)	6,140 (78.0)	116 (23.1)	110 (75.9)
	Mean # rx (95% CI)	1.3 (1.2-1.3)	*	1.7 (1.7-1.7)	2.1 (2.0-2.1)	1.5 (1.3-1.6)	2.1 (1.8-2.4)
	Mean days' supply, in days (95% CI)	8.6 (7.7-9.5)	*	20.3 (19.9-20.7)	51.3 (50.4-52.2)	24.2 (19.7-28.8)	53.7 (46.3-61.0)
	Mean daily dosage, in MME (95% CI)	34.3 (32.2-36.3)	*	32.4 (32.0-32.9)	43.5 (42.4-44.5)	25.4 (22.0-28.8)	39.4 (33.3-45.6)

[a] Prescribing rate, MME, days' supply, and number of prescriptions reflect all prescriptions supplied for that indication during the three months following the index diagnosis; thus, the prescribing rate is anchored to the number of *patients* with opioid prescriptions associated with each indication.
[b] Data are from the MarketScan Multi-State Medicaid Database, Q4 2016—Q3 2017.
[c] Patients not on long-term opioid therapy (LTOT) were those whose prescriptions did not meet the LTOT criteria.
[d] Long-term opioid therapy (LTOT) is defined as 1) having at least three opioid prescriptions in the three months prior to the index visit; 2) more than 60 total days of opioid supply; and 3) the gap between the end of one prescription and the next prescription was fewer than 10 days.
*****Too little data to report.

Abbreviations: Rx = prescription; MME = morphine milligram equivalents (available at https://www.cdc.gov/drugoverdose/resources/data.html); LTOT = long-term opioid therapy.

© 2020 Mikosz CA et al. *JAMA Network Open*.

eTable 9. Opioid Prescribing Rates and Amounts[a] for Postsurgical Pain Management Among Medicaid Enrollees Not on Long-term Opioid Therapy[b] in the United States, by Indication and Age Group, Q4 2016–Q3 2017[c,d]

Procedures	Age in Years	# Procedures with rx (%)	Days' Supply, in Days		Daily Dosage, in MME		Total # of procedures
			Mean (95% CI)	Median (Q1, Q3)	Mean (95% CI)	Median (Q1, Q3)	
Vaginal delivery	0-18	1,782 (28.0)	4.3 (4.2-4.4)	4.0 (3.0, 5.0)	37.7 (36.8-38.5)	33.3 (25.0, 45.0)	6,364
	19-64	33,426 (30.8)	4.2 (4.2-4.2)	4.0 (3.0, 5.0)	38.2 (38.0-38.4)	33.3 (25.0, 45.0)	108,443
Cesarean section	0-18	1,274 (83.9)	5.0 (4.9-5.1)	5.0 (3.0, 5.0)	48.3 (47.2-49.5)	45.0 (32.1, 56.3)	1,518
	19-64	41,286 (83.3)	5.1 (5.1-5.1)	5.0 (4.0, 6.0)	49.3 (49.1-49.5)	45.0 (32.1, 60.0)	49,559
Tonsillectomy	0-18	11,079 (33.2)	7.6 (7.5-7.6)	7.0 (5.0, 10.0)	23.2 (22.9-23.5)	18.8 (11.4, 30.0)	33,385
	19-64	1,719 (76.1)	6.7 (6.5-6.9)	6.0 (4.0, 8.0)	49.5 (48.4-50.7)	45.0 (30.0, 62.5)	2,259
Cholecystectomy, laparoscopic	0-18	1,423 (84.8)	4.6 (4.5-4.7)	4.0 (3.0, 5.0)	35.8 (34.9-36.6)	30.0 (21.4, 45.0)	1,679
	19-64	12,537 (88.1)	4.8 (4.8-4.8)	5.0 (3.0, 5.0)	42.9 (42.6-43.3)	37.5 (27.0, 52.5)	14,224
	65+	121 (74.2)	4.9 (4.3-5.4)	5.0 (3.0, 6.0)	43.9 (39.6-48.2)	41.9 (26.6, 50.0)	163
Sinus surgery	0-18	1,518 (37.0)	5.4 (5.3-5.6)	5.0 (4.0, 7.0)	31.9 (30.8-33.1)	30.0 (20.0, 40.0)	4,108
	19-64	4,363 (76.4)	5.2 (5.1-5.4)	5.0 (3.0, 6.0)	42.2 (41.4-42.9)	37.5 (28.6, 50.0)	5,713
	65+	24 (48.0)	7.1 (3.3-10.8)	5.0 (4.0, 7.0)	39.4 (27.6-51.3)	37.5 (30.0, 50.0)	50
Appendectomy, laparoscopic	0-18	3,000 (55.8)	4.2 (4.1-4.3)	4.0 (3.0, 5.0)	28.6 (28.1-29.2)	26.3 (18.8, 37.5)	5,381
	19-64	2,589 (79.9)	4.7 (4.6-4.8)	4.0 (3.0, 5.0)	45.3 (44.5-46.1)	42.5 (30.0, 56.3)	3,241
Spinal fusion	0-18	711 (66.4)	9.1 (8.7-9.4)	8.0 (6.0, 10.0)	41.1 (39.3-42.8)	37.5 (25.0, 46.9)	1,070
	19-64	2,397 (82.9)	9.1 (8.9-9.3)	8.0 (5.0, 10.0)	58.0 (56.8-59.2)	48.2 (30.0, 83.3)	2,890
	65+	28 (77.8)	6.5 (5.5-7.4)	6.0 (4.0, 10.0)	56.4 (44.4-68.3)	56.3 (28.6, 81.3)	36
Abdominal solid organ resection, laparoscopic	19-64	4,519 (92.5)	5.1 (5.0-5.2)	5.0 (3.0, 7.0)	46.7 (46.0-47.3)	45.0 (30.0, 60.0)	4,883
Total knee arthroplasty	19-64	2,609 (86.8)	8.3 (8.1-8.4)	7.0 (5.0, 10.0)	61.5 (60.3-62.7)	56.3 (31.3, 85.7)	3,007
	65+	143 (70.8)	9.0 (8.2-9.8)	7.0 (5.0, 10.0)	53.7 (49.0-58.4)	45.0 (30.0, 66.7)	202
Inguinal hernia repair, open	0-18	1,119 (44.6)	4.4 (4.2-4.6)	4.0 (3.0, 5.0)	15.6 (14.7-16.4)	10.0 (5.6, 21.0)	2,511
	19-64	1,410 (88.5)	5.4 (5.2-5.5)	5.0 (3.0, 7.0)	46.1 (45.0-47.2)	42.9 (30.0, 60.0)	1,593
	65+	51 (83.6)	6.4 (5.5-7.2)	5.0 (4.0, 9.0)	41.0 (35.3-46.8)	36.3 (25.0, 50.0)	61

© 2020 Mikosz CA et al. *JAMA Network Open*.

Procedures	Age in Years	# Procedures with rx (%)	Days' Supply, in Days		Daily Dosage, in MME		Total # of procedures
			Mean (95% CI)	Median (Q1, Q3)	Mean (95% CI)	Median (Q1, Q3)	
**Lumpectomy/partial mastectomy**	0-18	408 (76.5)	4.3 (4.1-4.6)	4.0 (3.0, 5.0)	32.9 (31.3-34.4)	30.0 (21.4, 40.0)	533
	19-64	1,647 (82.4)	4.8 (4.7-5.0)	5.0 (3.0, 6.0)	38.4 (37.5-39.3)	33.3 (25.0, 50.0)	1,998
	65+	18 (64.3)	5.1 (4.2-6.0)	5.0 (4.0, 7.0)	29.9 (23.8-36.0)	28.1 (20.0, 33.3)	28
**Combined spinal fusion and lumbar decompression surgery**	0-18	58 (51.8)	10.3 (8.2-12.5)	10.0 (5.0, 15.0)	43.6 (34.3-52.8)	43.0 (21.4, 50.6)	112
	19-64	1,716 (76.5)	8.9 (8.6-9.2)	8.0 (5.0, 10.0)	63.6 (61.0-66.1)	56.3 (33.3, 87.5)	2,242
	65+	22 (31.4)	6.1 (4.3-7.9)	7.0 (4.0, 8.0)	55.4 (32.8-78.0)	45.0 (20.0, 90.0)	70
**Arthroscopic knee surgery**	0-18	1,829 (90.8)	6.8 (6.6-6.9)	6.0 (5.0, 8.0)	52.8 (51.6-54.0)	45.0 (30.0, 75.0)	2,015
	19-64	1,192 (94.2)	6.8 (6.6-6.9)	6.0 (5.0, 8.0)	55.6 (54.2-57.0)	50.0 (30.0, 75.0)	1,265
**Lumbar decompression**	0-18	172 (51.0)	6.6 (6.0-7.2)	5.0 (4.0, 8.0)	28.8 (25.0-32.5)	21.7 (9.0, 42.9)	337
	19-64	1,793 (87.0)	8.2 (8.0-8.4)	7.0 (5.0, 10.0)	51.7 (50.5-52.8)	45.0 (30.0, 64.3)	2,061
	65+	22 (81.5)	8.0 (6.5-9.5)	7.0 (5.0, 10.0)	48.2 (36.1-60.2)	45.0 (20.0, 60.0)	27
**Excisional biopsy**	0-18	242 (35.6)	4.9 (4.4-5.3)	4.0 (3.0, 6.0)	25.1 (23.3-26.9)	22.5 (15.0, 30.0)	679
	19-64	789 (56.6)	5.1 (4.9-5.4)	5.0 (3.0, 7.0)	41.0 (39.5-42.6)	33.3 (25.0, 50.0)	1,395
	65+	20 (41.7)	7.4 (4.7-10.0)	5.0 (4.0, 10.0)	41.4 (29.2-53.5)	32.1 (20.0, 56.3)	48
**Total hip arthroplasty**	0-18	21 (100.0)	10.7 (7.8-13.6)	10.0 (7.0, 14.0)	41.5 (33.0-49.9)	37.5 (30.0, 53.1)	21
	19-64	1,672 (83.6)	8.4 (8.2-8.6)	7.0 (5.0, 10.0)	62.1 (60.4-63.8)	56.3 (31.3, 85.7)	2,001
	65+	52 (74.3)	7.6 (6.4-8.9)	6.0 (4.0, 10.0)	52.4 (45.1-59.6)	45.0 (30.0, 75.0)	70
**Arthroscopic rotator cuff repair**	0-18	19 (70.4)	5.9 (5.0-6.9)	5.0 (5.0, 7.0)	50.8 (39.1-62.5)	41.3 (30.0, 75.0)	27
	19-64	1,781 (94.7)	7.3 (7.1-7.4)	7.0 (5.0, 10.0)	56.5 (55.4-57.7)	50.0 (30.0, 75.0)	1,880
	65+	37 (94.9)	8.1 (6.3-9.9)	6.0 (5.0, 10.0)	53.8 (46.3-61.3)	57.5 (30.0, 68.3)	39
**Parathyroid or thyroid surgery**	0-18	72 (64.3)	4.1 (3.6-4.6)	4.0 (2.0, 5.0)	31.2 (26.9-35.6)	30.0 (18.8, 37.5)	112
	19-64	1,172 (83.4)	5.2 (5.0-5.4)	5.0 (3.0, 7.0)	43.9 (42.6-45.1)	40.0 (30.0, 50.0)	1,406
	65+	23 (74.2)	5.7 (4.4-7.0)	5.0 (4.0, 7.0)	38.1 (29.5-46.6)	33.3 (30.0, 48.8)	31
**Colectomy, open**	0-18	25 (15.2)	6.6 (5.0-8.2)	5.0 (4.0, 8.0)	31.9 (24.6-39.2)	25.0 (19.3, 45.0)	164
	19-64	383 (39.2)	5.4 (5.2-5.7)	5.0 (3.0, 7.0)	50.5 (47.2-53.9)	42.9 (30.0, 60.0)	977

© 2020 Mikosz CA et al. *JAMA Network Open*.

Procedures	Age in Years	# Procedures with rx (%)	Days' Supply, in Days		Daily Dosage, in MME		Total # of procedures
			Mean (95% CI)	Median (Q1, Q3)	Mean (95% CI)	Median (Q1, Q3)	
**Inguinal hernia repair, laparoscopic**	0-18	177 (49.7)	3.8 (3.5-4.1)	3.0 (2.0, 5.0)	22.0 (19.3-24.8)	15.0 (7.5, 30.0)	356
	19-64	843 (90.3)	5.4 (5.2-5.6)	5.0 (4.0, 7.0)	46.7 (45.2-48.1)	45.0 (30.0, 60.0)	934
	65+	20 (83.3)	6.0 (5.1-7.0)	6.0 (5.0, 8.0)	39.8 (31.2-48.5)	37.5 (30.0, 42.9)	24
**Coronary artery bypass**	0-18	26 (40.6)	3.9 (2.3-5.4)	3.0 (2.0, 5.0)	25.5 (17.7-33.3)	22.5 (7.9, 37.5)	64
	19-64	417 (40.3)	7.3 (6.9-7.7)	7.0 (5.0, 9.0)	48.4 (45.4-51.3)	42.9 (30.0, 60.0)	1,035
	65+	20 (31.3)	7.3 (5.9-8.7)	8.0 (5.0, 10.0)	42.6 (34.4-50.7)	45.0 (31.3, 56.3)	64
**Simple mastectomy**	0-18	44 (88.0)	5.6 (4.7-6.5)	5.0 (3.0, 7.0)	30.5 (26.7-34.3)	30.0 (21.4, 34.2)	50
	19-64	283 (87.1)	5.5 (5.1-5.9)	5.0 (4.0, 7.0)	53.1 (49.7-56.6)	45.0 (32.1, 75.0)	325
	65+	11 (91.7)	8.3 (1.7-15.0)	5.0 (4.0, 10.0)	25.8 (18.1-33.5)	30.0 (21.4, 30.0)	12
**Colectomy, laparoscopic**	0-18	26 (42.6)	4.7 (3.8-5.7)	5.0 (3.0, 6.0)	31.8 (26.5-37.1)	30.0 (21.4, 45.0)	61
	19-64	369 (67.6)	5.5 (5.3-5.8)	5.0 (4.0, 7.0)	51.6 (48.6-54.5)	45.0 (30.0, 62.5)	546
**Cholecystectomy, open**	0-18	17 (47.2)	4.5 (3.5-5.5)	4.0 (3.0, 6.0)	32.1 (24.8-39.4)	30.0 (15.0, 45.0)	36
	19-64	212 (60.6)	5.3 (4.9-5.6)	5.0 (3.0, 7.0)	46.3 (43.2-49.3)	40.0 (28.1, 60.0)	350

[a] Reported outcome data (prescribing rate, MME, and days' supply) reflect prescriptions supplied for a specific procedure or visit, meaning that the prescribing rate is anchored to visits/procedures.
[b] Patients not on long-term opioid therapy (LTOT) were those whose prescriptions did not meet the LTOT criteria. LTOT is defined as 1) having at least three opioid prescriptions in the three months prior to the index visit; 2) more than 60 total days of opioid supply; and 3) the gap between the end of one prescription and the next prescription was fewer than 10 days.
[c] Data are from the MarketScan Multi-State Medicaid Database, Q4 2016—Q3 2017.

[d] Not all age groups are well-represented for every procedure and are thus not listed here.

Abbreviations: Rx = prescription; MME = morphine milligram equivalents (available at https://www.cdc.gov/drugoverdose/resources/data.html). (Q1, Q3) represent the interquartile range.

© 2020 Mikosz CA et al. *JAMA Network Open*.

eReferences

1) Tian TY, Zlateva I, Anderson DR. Using electronic health records data to identify patients with chronic pain in a primary care setting. J Am Med Inform Assoc 2013;20:e275–80.

2) Romanelli R, Shah S, Ikeda L et al. Patient characteristics and healthcare utilization of a chronic pain population within an Integrated Healthcare System. Am J Manag Care. 2017;23(2):e50—e56

3) Snyder AB, Zhou M, Theodore R, Quarmyne MO, Eckman J, Lane PA. Improving an Administrative Case Definition for Longitudinal Surveillance of Sickle Cell Disease. Public Health Rep. 2019 May/Jun;134(3):274-281

4) Reeves S, Garcia E, Kleyn M, Housey M, Stottlemyer R, Lyon-Callo S, Dombkowski KJ. Identifying sickle cell disease cases using administrative claims. Acad Pediatr. 2014 Sep-Oct;14(5 Suppl):S61-7.

5) Robbins MS, Starling AJ, Pringsheim TM, Becker WJ, Schwedt TJ. Treatment of Cluster Headache: The American Headache Society Evidence-Based Guidelines. Headache. 2016 Jul;56(7):1093-106

6) Dowell D, Haegerich TM, Chou R. CDC guideline for prescribing opioids for chronic pain – United States, 2016. Morbidity and Mortality Weekly Report Recommendations and Reports 2016;65:1-49.

7) American College of Occupational and Environmental Medicine. Opioids, 2017. Published by the Reed Group.

8) Qaseem A, Wilt TJ, McLean RM, Forciea MA; Clinical Guidelines Committee of the American College of Physicians. Noninvasive Treatments for Acute, Subacute, and Chronic Low Back Pain: A Clinical Practice Guideline From the American College of Physicians. Ann Intern Med. 2017 Apr 4;166(7):514-530. doi: 10.7326/M16-2367. Epub 2017 Feb 14.

9) Washington State Agency Medical Director's Group. Interagency Guideline on Prescribing Opioids for Pain, 2015. Retrieved from http://www.agencymeddirectors.wa.gov/Files/2015AMDGOpioidGuideline.pdf

10) National Heart, Lung, and Blood Institute, National Institutes of Health. Evidence-Based Management of Sickle Cell Disease: Expert Panel Report, 2014. Accessed from: http://www.nhlbi.nih.gov/guidelines.

© 2020 Mikosz CA et al. *JAMA Network Open*.
